# MMP-9/Gelatinase B Degrades Immune Complexes in Systemic Lupus Erythematosus

**DOI:** 10.3389/fimmu.2019.00538

**Published:** 2019-03-22

**Authors:** Estefania Ugarte-Berzal, Lise Boon, Erik Martens, Vasily Rybakin, Daniel Blockmans, Jennifer Vandooren, Paul Proost, Ghislain Opdenakker

**Affiliations:** ^1^Laboratory of Immunobiology, Rega Institute for Medical Research, KU Leuven, Leuven, Belgium; ^2^Department of General Internal Medicine, University Hospital Gasthuisberg, KU Leuven, Leuven, Belgium; ^3^Laboratory of Molecular Immunology, Rega Institute for Medical Research, Department of Microbiology and Immunology, KU Leuven, Leuven, Belgium

**Keywords:** matrix metalloproteinase-9, immune complexes, immunoglobulins, systemic lupus erythematosus, drug-induced lupus erythematosus

## Abstract

Systemic Lupus Erythematosus (SLE) is a common and devastating autoimmune disease, characterized by a dysregulated adaptive immune response against intracellular antigens, which involves both autoreactive T and B cells. In SLE, mainly intracellular autoantigens generate autoantibodies and these assemble into immune complexes and activate the classical pathway of the complement system enhancing inflammation. Matrix metalloproteinase-9 (MMP-9) levels have been investigated in the serum of SLE patients and in control subjects. On the basis of specific studies, it has been suggested to treat SLE patients with MMP inhibitors. However, some of these inhibitors induce SLE. Analysis of LPR^−/−^MMP-9^−/−^ double knockout mice suggested that MMP-9 plays a protective role in autoantigen clearance in SLE, but the effects of MMP-9 on immune complexes remained elusive. Therefore, we studied the role of MMP-9 in the clearance of autoantigens, autoantibodies and immune complexes and demonstrated that the lack of MMP-9 increased the levels of immune complexes in plasma and local complement activation in spleen and kidney in the LPR^−/−^ mouse model of SLE. In addition, we showed that MMP-9 dissolved immune complexes from plasma of lupus-prone LPR^−/−^/MMP-9^−/−^ mice and from blood samples of SLE patients. Surprisingly, autoantigens incorporated into immune complexes, but not immunoglobulin heavy or light chains, were cleaved by MMP-9. We discovered Apolipoprotein-B 100 as a new substrate of MMP-9 by analyzing the degradation of immune complexes from human plasma samples. These data are relevant to understand lupus immunopathology and side-effects observed with the use of known drugs. Moreover, we caution against the use of MMP inhibitors for the treatment of SLE.

## Introduction

SLE is a chronic and complex systemic autoimmune disease, which affects all major organ systems. In SLE the autoantigens (aAg) are typically ubiquitous intracellular proteins and protein-nucleic acid complexes. This multi-system disease is characterized by the production of non-organ-specific, self-reactive autoantibodies (aAb) directed against these intracellular components, for example DNA, RNA, and ubiquitous proteins such as p53, actins, tubulins, and histones (1, 2). In fact, more than 180 different aAb have been found in SLE patients (3). The presence of aAb leads to the formation of immune complexes (IC) which are detectable in the circulation. IC deposition occurs by hemoconcentration or other hemodynamic forces at specific anatomical sites and activates locally the complement system. This causes much of the tissue damage observed in, e.g., the kidneys, skin, lungs, and joints of SLE patients and leads to health problems, such as increased infection rates, renal and skin disorders, neurological complications, fibromyalgias, osteoporosis, rheumatoid arthritis, and osteoarthritis (2).

The origin of the aAg and the subsequent generation of aAb and IC in SLE is presently explained by a high apoptosis rate and by a defect in the clearance of apoptotic cells and neutrophil extracellular traps in SLE patients (4, 5). Once IC have been formed, these are normally cleared by the complement and the macrophage phagocytic systems and defects in some of these processes have been described in SLE patients.

Some forms of SLE are provoked by the use of drugs, this type of SLE is called drug-induced lupus erythematosus (DILE). Normally, DILE is resolved within days to months after withdrawal of the culprit drug in those patients with no underlying immune system dysfunction (6–8).

The treatment of patients with SLE without major organ manifestation is with anti-inflammatory glucocorticoids and antimalarial drugs, and often it is a pure symptomatic treatment (9). The used drugs display side-effects and toxicity and do not specifically target aAg-IC *per se*. Some specific strategies target immune complex formation by reducing antibody production (targeting B cells), reducing the binding of aAb, reducing the availability of nucleosome material, increasing the clearance of IC, and interfering with feedback loops (10). However, none of these strategies succeeded in complete inhibition of IC formation. Therefore, further research is necessary to understand the basic mechanisms that trigger IC formation and to improve treatments.

An important protease studied in inflammation, also in SLE, is gelatinase B/matrix metalloproteinase-9 (MMP-9). MMP-9 is secreted by different cell types, including mainly neutrophils, but also and to a lesser extent by activated macrophages, T and B lymphocytes, endothelial cells, and smooth muscle cells (11). MMP-9 cleaves many molecules (11–13) including, intracellular proteins (13). Numerous of these substrates are aAg in SLE (12).

The role of MMP-9 as a detrimental or beneficial molecule also remains an unanswered question in SLE, due to discrepancies in the published data. Several authors report higher serum levels of MMP-9 in SLE patients compared with those of healthy controls, whereas others do not detect significant differences (14–19). Surprisingly, no correlation exists between serum MMP-9 levels and the number of peripheral blood cells in SLE patients (20) and the levels of MMP-9 in the circulation inversely correlate with the amounts of anti-dsDNA antibodies (21) which is an indication of the severity of the disease.

Due to the unsolved role of MMP-9 in SLE, and because some MMP-inhibitory drugs are able to induce DILE, it is clinically relevant to analyze the role of MMP-9 in this disease. Therefore, we studied the role of MMP-9 in SLE by comparing the lpr loss-of-function mutation in the apoptosis mediator fas knockout mice on a C57Bl/6 background (LPR^−/−^ mice) with mice lacking both MMP-9 and functional apoptosis receptor Fas (LPR^−/−^MMP-9^−/−^ mice). In previous work, it was shown that LPR^−/−^ animals develop a moderate SLE-like syndrome (22), whereas the double knockout mice lacking MMP–9 present reduced survival rates, extreme lymphadenopathy and splenomegaly and increased aAb production and therefore, pronounced autoimmune tissue injury (23).

Here, we specifically studied the role of MMP-9 in the cleavage of auto-IC. We showed that switching MMP-9 off in the SLE mouse model LPR^−/−^ results in higher levels of IC in the circulation and spleen and kidney. We proved that MMP-9 does not cleave immunoglobulins but destroys autoantigens captured in IC. We studied the effects of active MMP-9 on IC degradation, by purifying IC from plasma samples from SLE mice with various degrees of SLE-like diseases, as well as from SLE patients. Serendipitously, this led to the discovery of a new substrate of MMP-9. Finally, with the use of an air-pouch model in WT and MMP-9^−/−^ mice, we studied *in vivo* the role and the efficiency of MMP-9 in IC clearance. Collectively, all our data are in line with the thesis that MMP-9 plays essential roles in preventing the formation of IC and in IC clearance.

## Results

### The Levels of IC Are Higher in the SLE Mouse Model When MMP-9 Is Genetically Deleted

**Figure 1:**
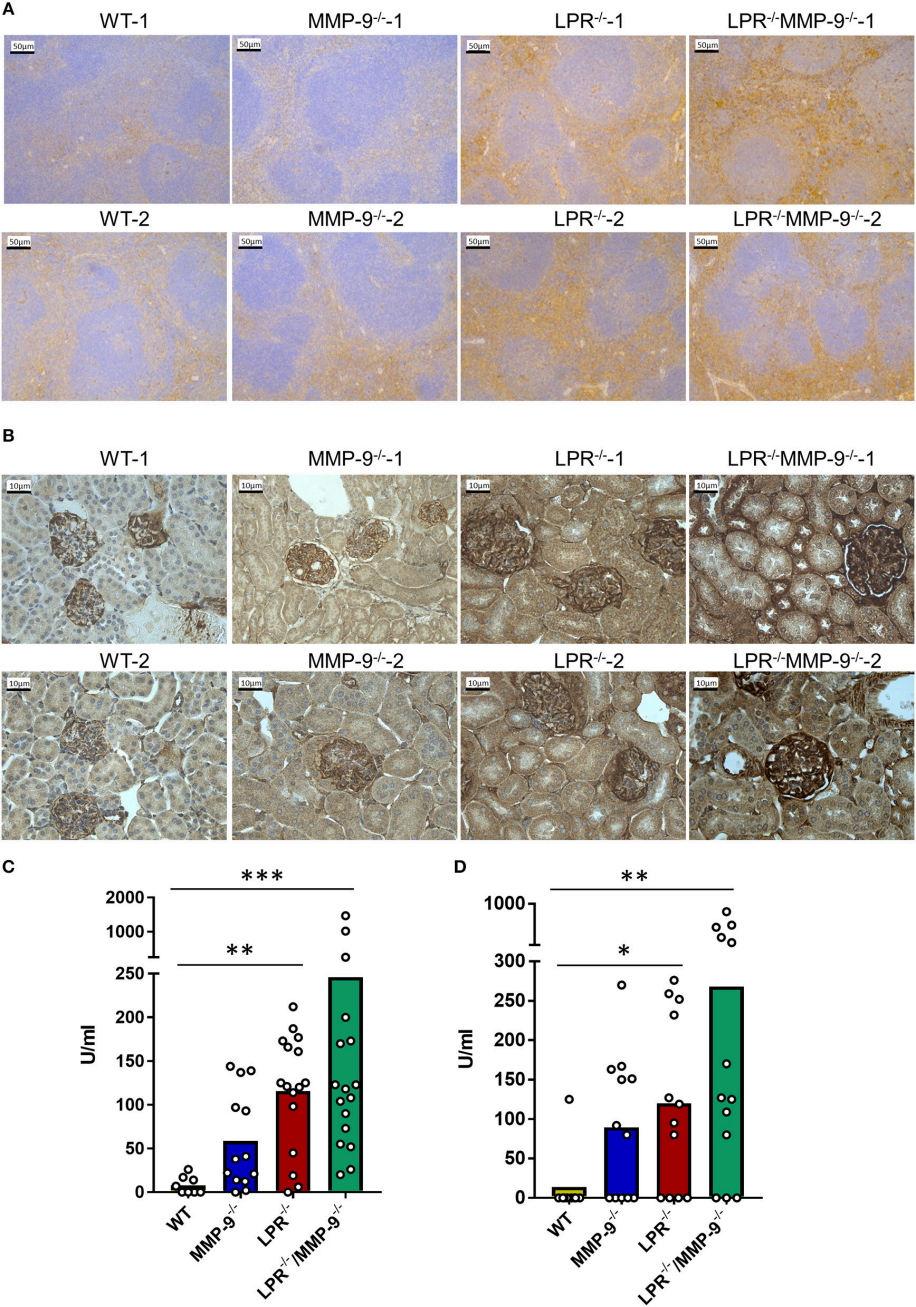
Levels of C3d in spleens and kidneys from WT, MMP-9^−/−^, LPR^−/−^, LPR^−/−^/MMP-9^−/−^ mice. **(A,B)** Immunohistochemical analysis of C3d as read-out of classical IC-mediated complement activation in WT, MMP-9^−/−^, LPR^−/−^, and LPR^−/−^/MMP-9^−/−^ spleens **(A)** and kidneys **(B)**. The horizontal bars in the images represent 50 μm for a magnification of 20 times for spleens and 10 μm for a magnification of 40 times for kidneys. Brown staining represents semi-quantitatively the level of complement factor C3d. **(C,D)** Anti C3d ELISAs of spleen tissue extracts **(C)** from 9 WT, 14 MMP-9^−/−^, 16 LPR^−/−^ and 17 LPR^−/−^/MMP-9^−/−^ mice and of kidney tissue extracts **(D)** of 13 WT, 16 MMP-9^−/−^, 16 LPR^−/−^, and 17 LPR^−/−^/MMP-9^−/−^ mice. *P*-values were determined by ANOVA Kruskal-Wallis test. ^*^p < 0.05, ^**^p < 0.01, and ^***^p < 0.001. Throughout the manuscript the following color code was used: yellow=WT, blue-=MMP-9^−/−^, red=LPR^−/−^ and green=LPR^−/−^/MMP-9^−/−^.

The spleen is one of the main filter organs in charge of IC clearance (24) and kidneys are typical organs affected in SLE due IC deposition (5). We evaluated the deposition of IC in the spleen and kidney of single LPR^−/−^ and in double knock out LPR^−/−^MMP-9^−/−^ mice by analyzing local complement activation with the use of C3d immunostaining. The signal from C3d was increased in the spleens and kidneys from LPR^−/−^ mice in comparison with WT organs. Moreover, genetic knock out of MMP-9 in this lupus mouse model (LPR^−/−^MMP-9^−/−^) led to further increases of complement activation in the spleen and kidney (Figures 1A,B and Supplemental Figures 1–10). In addition, we measured the levels of C3d in tissue extracts of spleen and kidneys by sandwich ELISA (Figures 1C,D). Whereas, significantly higher C3d levels were observed in the comparisons of WT and LPR^−/−^ mice and between WT and LPR^−/−^/MMP-9^−/−^ mice, the deletion of the MMP-9 gene only yielded trends toward higher C3d levels. With these data, we were stimulated to study further the role of MMP-9 in IC clearance or avoidance of IC deposition in WT animals.

**Figure 2:**
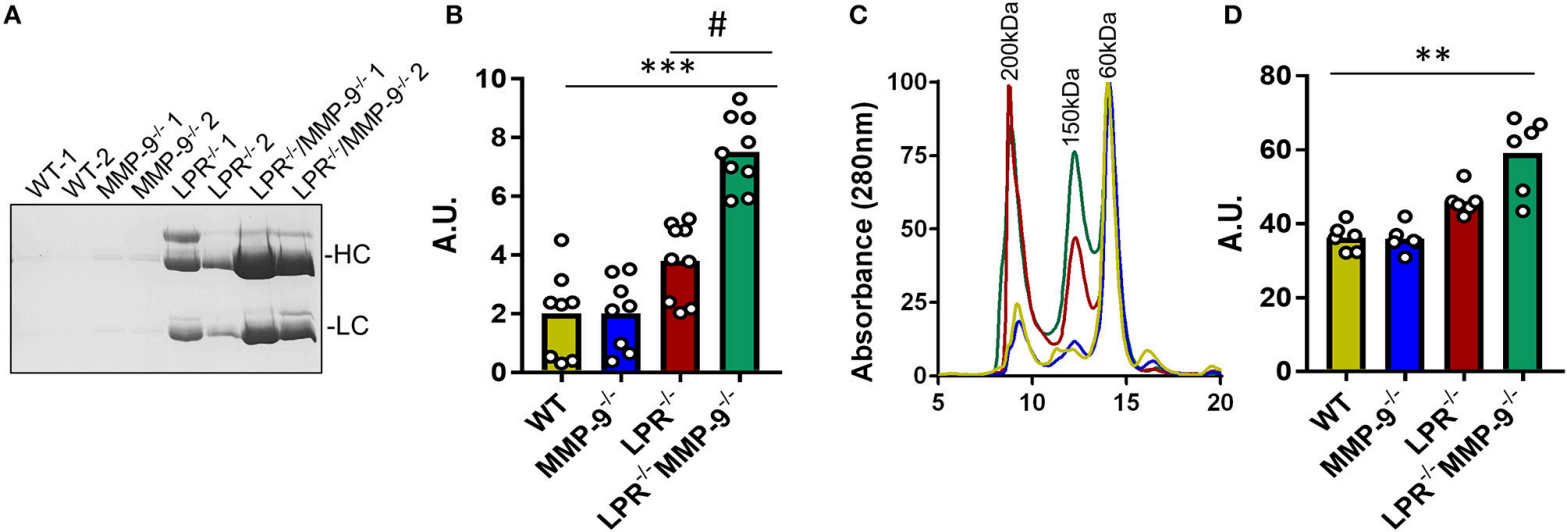
Levels of immune complexes in plasma samples from WT, MMP-9^−/−^, LPR^−/−^, LPR^−/−^/MMP-9^−/−^ mice. **(A)** SDS-PAGE and Coomassie blue staining of the IC proteins purified from plasma samples of two WT, MMP-9^−/−^, LPR^−/−^ and LPR^−/−^/MMP-9^−/−^ mice. HC indicates heavy chains and LC represents light chains. **(B)** Every dot represents a sample analysis from an individual mouse. Quantification of the heavy chains from the IC shown in **(A)** and Supplemental Figure 11 with the use of ImajeJ software. A. U. = arbitrary units by quantification of Numbers were obtained by quantification of heavy chain signals with ImajeJ software. **(C)** Averaged gel filtration chromatography profiles of plasma proteins (absorbance at 280 nm) of the four genotypes studied at 9 months. Details of individual profiles are shown in Supplemental Figure 12. Significant differences in protein levels are clear and comparison with samples at 3 months are provided in Supplemental Figure 13. **(D)** Quantification of the area under the peak of 150 and 200 kDa which represent Ab and IC. A.U = Arbitrary units of the peak area obtained with the Unicorn 5.1 software. *P*-values were determined by ANOVA Kruskal-Wallis test. ^**^p < 0.01, ^***^p < 0.001 for the comparisons of WT and LPR^−/−^/MMP-9^−/−^ mice and #*p* < 0.05 for comparisons of LPR^−/−^ and LPR^−/−^/MMP-9^−/−^ mice.

By purification of the IC from plasma samples and analysis by SDS-PAGE we showed that the abundance of IC was higher in double knock out (LPR^−/−^MMP-9^−/−^) than in the single LPR^−/−^ knockout mice (Figures 2A,B and Supplemental Figure 11). We also performed gel filtration chromatography analysis of plasma samples derived from the four mouse genotypes studied. The profiles of the chromatographic analyses contained three main peaks at approximately 60, 150, and 200 kDa, which corresponded with albumin, antibodies, and large molecules/complexes, respectively. The gel filtration chromatography profiles indicated that the levels of molecules larger than 150 kDa and corresponding to the IC, were more abundant in the SLE mouse model LPR^−/−^ than in the WT and MMP-9^−/−^ controls. In addition, the levels of these high molecular weight proteins further increased in the absence of MMP-9 (LPR^−/−^MMP-9^−/−^) (Figures 2C,D and Supplemental Figure 12). In line with these experiments, we studied the development and increases of IC in the two SLE mouse models (LPR^−/−^ and LPR^−/−^MMP-9^−/−^) as a function of time, and observed that the levels of IC were not elevated at 3 months in the SLE mouse models, but increased gradually with time and, consequently, with the progression of the disease (Supplemental Figure 13). The profiles of the plasma proteins from the four genotypes were similar at 3 months, whereas at 9 months the plasma of the LPR^−/−^ mice and, to a higher degree, of the LPR^−/−^MMP-9^−/−^ mice, contained increased levels of IC and antibodies. We concluded that the lack of MMP-9 correlates with increased IC plasma levels and possibly deposition in spleens and kidneys.

### actMMP-9 Does Not Cleave Immunoglobulins IgG and IgM

**Figure 3:**
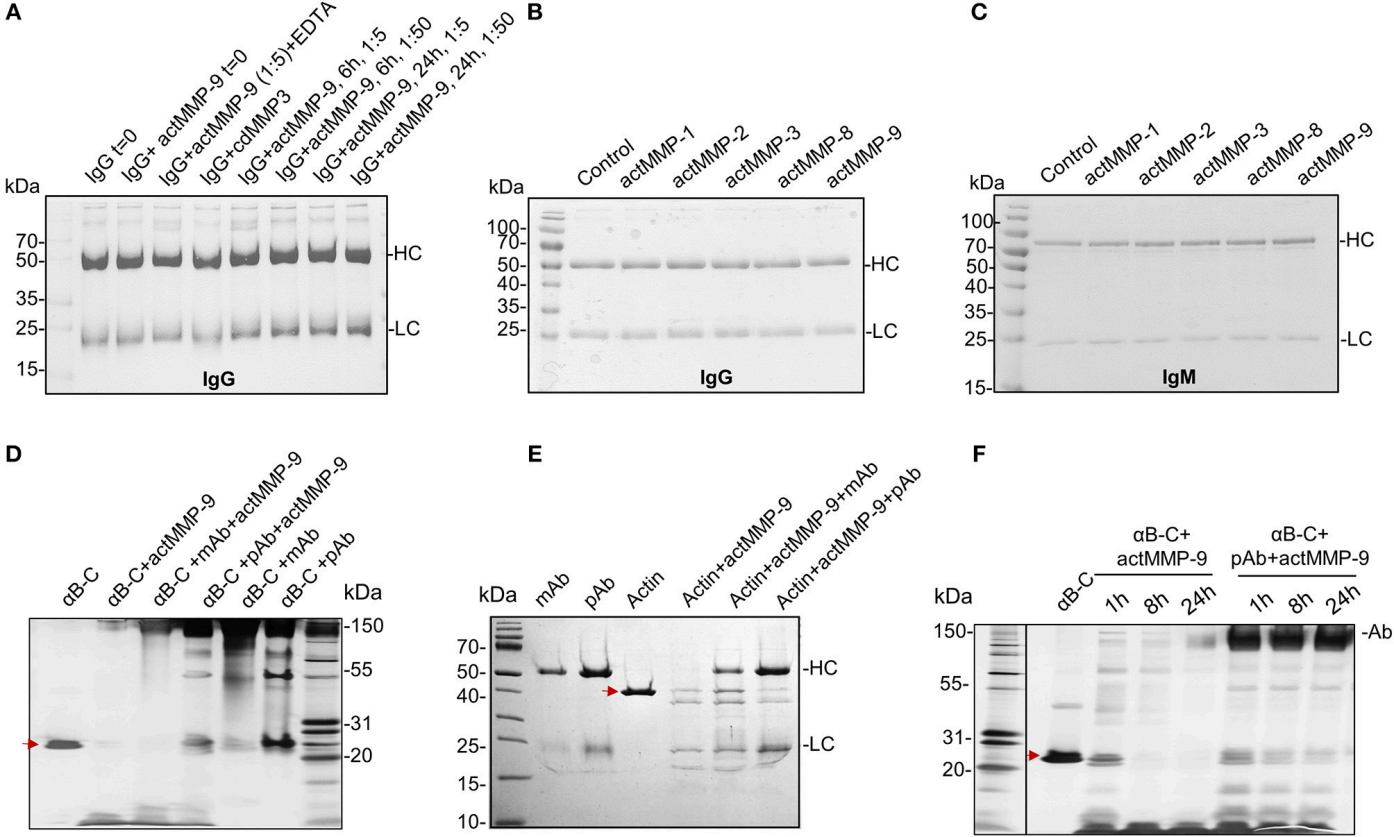
actMMP-9 does not degrade IgG and IgM but cleaves αB-crystallin and actin in IC. **(A)** IgG incubated with the indicated proteases and/or inhibitor EDTA. After 0, 6, and 24 h, the products of the incubation were analyzed by SDS-PAGE and Coomassie Blue staining of residual proteins. **(B,C)** IgG **(B)** or IgM **(C)** immunoglobulins were incubated with the indicated active MMPs during 24 h. The proteins in the SDS-PAGE gels were stained with Coomassie Blue. HC, Heavy chain; LC, Light chain. **(D,E)** αB-crystallin (αB-C, red arrow in **D**) or actin (red arrow in **E**), in free form or within IC with polyclonal (pAb) or monoclonal antibody (mAb) incubated with actMMP-9 at 37°C. After 24 h the proteins were separated by SDS-PAGE and analyzed by silver staining. **(F)** Free αB-crystallin (red arrow) or in a pAb-IC incubated with actMMP-9 for 1, 8 or 24 h at 37°C. After incubation, SDS-PAGE separation and silver staining analysis of the proteins were performed.

MMP-9 has been shown to cleave organ-specific aAg, such as myelin basic protein in multiple sclerosis (25), collagen in arthritis (26), insulin in diabetes (27), and ubiquitous aAg in systemic autoimmune diseases (12, 23). Therefore, it was relevant to investigate whether these cleavages also occur with aAg captured within IC. Because immunoglobulins (Ig) are much larger proteins (more than 150 kDa) than most of the named aAg, a first logical step toward this study was to investigate whether MMP-9 cleaves Ig, in particular IgG, a major antibody in SLE-related IC. Human IgG was incubated with active MMP-9 (actMMP-9) at 37°C during 6 or 24 h at a ratio enzyme: Ig of 1:5 or 1:50. The products of the incubation were then analyzed by SDS-PAGE. Figure 3A shows that neither the heavy chain (HC) nor the light chain (LC) of IgG were cleaved under any of the conditions analyzed. To further study the role of MMPs in degrading Igs, we incubated IgG or IgM (as the 2 Ig classes activating the classical complement system) with actMMP−1,−2,−3−8, and−9 at a ratio enzyme:Ig 1:1 during 24 h. Again and in contrast with our expectations, none of the incubations resulted in IgG or IgM degradation by any of the MMPs studied (Figures 3B,C and Supplemental Figures 14A,B). Obviously, a strong natural selection pressure exists against proteolysis of IgG and IgM by MMP-9.

### αB-Crystallin and Actin Within IC Are Cleaved by MMP-9

We next investigated whether actMMP-9 degrades known substrates like αB-crystallin and actin (12), when embedded within IC. First, αB-crystallin and actin were incubated with their respective polyclonal or monoclonal antibodies in a ratio Ab:antigen 2:1 during 30′ at room temperature to generate IC (Figures 3D,E and Supplemental Figures 14C,D). These IC or the antigens alone were incubated with actMMP-9 during 1 h. αB-crystallin alone or in IC was cut by MMP-9, but the results of the cleavages were different when the IC were formed with a polyclonal (pAb) or a monoclonal antibody (mAb). Figure 3D and Supplemental Figure 14D shows that the migration patterns of cleavage products (generated by actMMP-9) of αB-crystallin alone or the IC with a mAb were similar. However, when the IC of αB-crystallin were formed with a pAb, the cleavage of the antigen was consistently inhibited. A different result was obtained with actin as antigen. Actin-IC with pAb or mAb resulted in similar cleaved actin fragments after incubation with actMMP-9 (Figure 3E and Supplemental Figure 14C).

Next, we studied if the protection against the cleavage caused by the pAb reactive with αB-crystallin was maintained for a long incubation time interval. In Figure 3F and Supplemental Figure 14E we showed that the cleavage of αB-crystallin by MMP-9 increased with incubation time and, after 24 h, only a small band with remnant epitopes of about 10 kDa was remaining. The incubation of pAb-αB-crystallin IC with actMMP-9 was much less affected, suggesting that the αB-crystallin cleavage sites were protected by the pAb.

These results were in line with the thesis that actMMP-9 was able to degrade protein antigens in IC, but that the efficiency of the cleavage depended on the ability of the Ab to mask the cleavage site(s).

### actMMP-9 Cleaves Mouse SLE Autoantigens in IC

**Figure 4:**
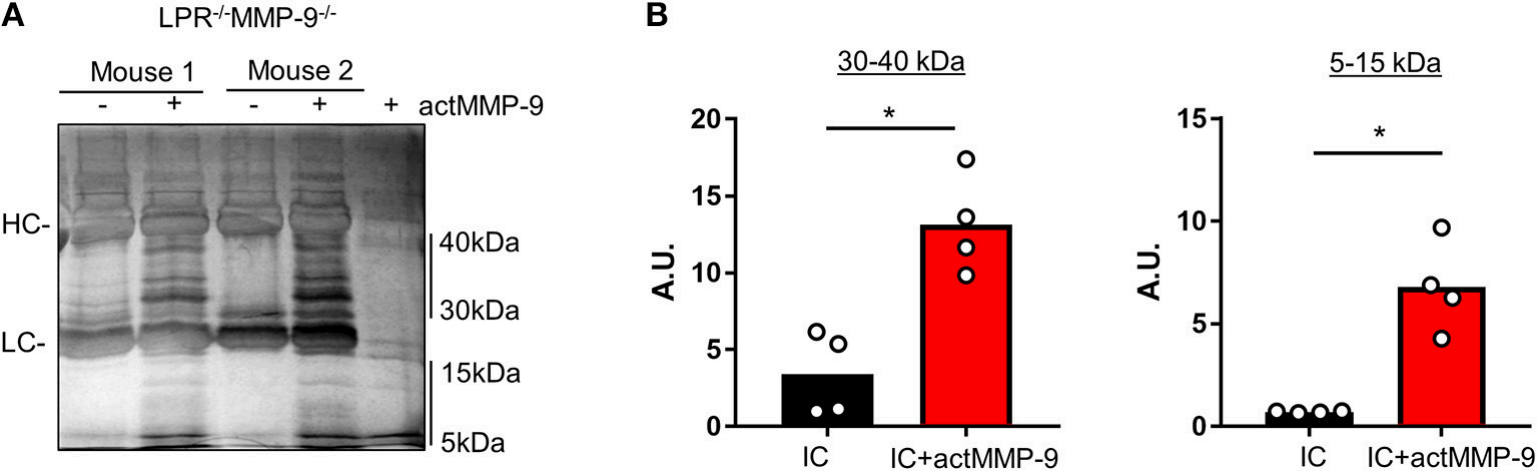
Cleavage of IC from plasma of LPR^−/−^MMP-9^−/−^ mice by actMMP-9. **(A)** IC from LPR^−/−^MMP-9^−/−^ mice were incubated with actMMP-9 at 37°C, 24 h. The products of the incubations were analyzed by SDS-PAGE and silver staining of residual proteins. **(B)** Quantification by densitometry of the proteins between 30 to 40 kDa and 5 to 15 kDa of 4 LPR^−/−^/MMP-9^−/−^ mice. *P*-values were determined by ANOVA Kruskal-Wallis test. ^*^p < 0.05. HC, Heavy chain; LC, Light chain. A.U, arbitrary units representing the densitometry analysis of the proteins with the use of ImajeJ software.

We then studied if actMMP-9 was able to degrade circulating IC from LPR^−/−^MMP-9^−/−^ mice. The results in Figure 4A showed that IC incubated with actMMP-9 generated different protein banding patterns, resulting from antigen digestion by MMP-9. Again, the immunoglobulin heavy and light chains persisted after proteolysis. However, actMMP-9 generated many remnant proteins/peptides within the molecular ranges of 30–40 kDa and 5–15 kDa, respectively. The densitometric quantification of the proteins between 30 and 40 kDa and between 5 and 15 kDa were in line with the possible role of MMP-9 in dissolving IC in the SLE mouse model (Figure 4B).

### actMMP-9 Cleaves Autoantigens in IC and Dissolves Human SLE IC

**Figure 5:**
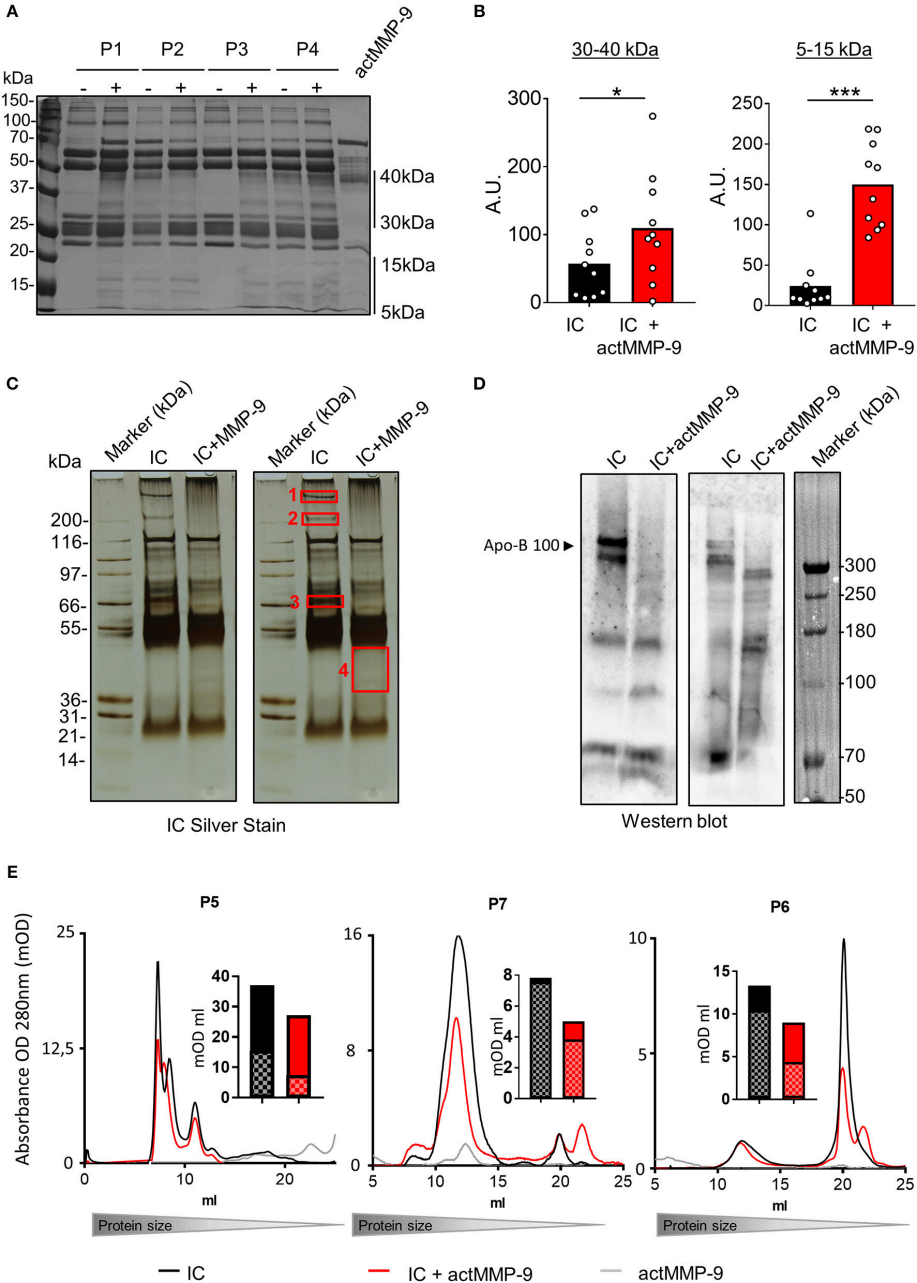
IC in plasma from SLE patients are cleaved by actMMP-9. **(A)** IC purified from plasma samples of SLE patients incubated in the presence or absence of actMMP-9 and analyzed by SDS-PAGE and silver staining (representative gel). **(B)** Quantification of the protein patterns between 30 to 40 kDa and 5 to 15 kDa from the experiments as shown in **(A)**. **(C)** Similar experiment as in **(A)** with a plasma IC sample from a different patient (P5). The proteins contained within red rectangles (1–4) on the duplicated photograph were sliced out of the gel for trypsin digestion and protein identification. **(D)** Western blot analysis for Apo-B 100 of IC from two donors after incubation with actMMP-9 at 37°C for 24 h. **(E)** Gel filtration chromatography profiles of IC purified from 3 patients (P5–P7) incubated in the presence or absence of actMMP-9. The histograms in full black and full red (mOD/ml) represent the areas of the total surface under the gel filtration chromatography profiles of IC alone and IC+actMMP-9, respectively. The histogram sections with squares and color tones design represent the quantification of the area for the major (IC) chromatography peaks. *P*-values were determined by ANOVA Kruskal-Wallis test. ^*^p < 0.05 and ^***^p < 0.001. A.U. = arbitrary representing the densitometry analysis of the proteins with the use of ImajeJ software.

Next, we studied whether actMMP-9 degraded human purified IC from SLE patients. Information of the 10 SLE patients is provided in Supplemental Table 1. In analogy with the preparation and analysis of plasma from the SLE mouse models, we processed the human IC samples in a similar way. Purified IC from individual SLE patients (P1–P10) were incubated with actMMP-9 at 37°C. After 16 h, the products of the incubations were separated by SDS-PAGE and silver-stained. The predominant protein bands in these preparations consisted of the intact heavy chain (HC) and light chain (LC) of human Igs as it is shown in the example gel in Figure 5A. After the incubation of IC with actMMP-9, different banding patterns of the proteins were observed, suggesting that MMP-9 cleaved some antigens within the IC (Figure 5A). The changes observed in the banding patterns between 30 and 40 kDa and between 5 and 15 kDa, were analyzed and quantified (Figure 5B).

We next investigated which proteins were present in the bands detected after actMMP-9 incubation. To this end, we sliced out the four indicated proteins bands in Figure 5C and we performed trypsin digestion followed by nanoLC/TOF/MS and protein identification. The obtained results indicated the presence of already known MMP-9 substrates, including C4, fibronectin and C1q (Supplemental Table 2). Interestingly, autoantibodies against these proteins have been found in SLE (3). In addition, a novel MMP-9 substrate in band 1 was identified: Apolipoprotein B 100 (Apo-B 100). To corroborate that Apo-B 100 was a new substrate of MMP-9, we purified IC from plasma samples and we performed Western blot analysis for Apo-B 100. After incubation with actMMP-9 the signal of intact Apo-B 100 disappeared, demonstrating that actMMP-9 cleaved Apo-B 100 (Figure 5D). As an internal control, we also evaluated appearing proteins/peptides after cleavage (Figure 5C, band 4). In this case, we identified sequences of the fibrinogen alpha chain, a known substrate of MMP-9, and of MMP-9 itself.

To study if the cleavage of the aAg in IC resulted in a change of the IC composition, we also performed gel filtration chromatography analysis of the IC from plasma of 3 SLE patients (P5, P6, P7), before and after incubation with actMMP-9. With this method, we analyzed the size of the IC under native physiological conditions in solution (Figure 5E). After incubation with actMMP-9, the profiles of the IC were altered in comparison with the intact non-incubated IC for P5 and P7 and to a lesser extent for P6. The main peak that corresponded to the IC was consistently reduced: 52, 49, and 41% for P5, P7, and P6, respectively. Collectively, this suggested that MMP-9 degraded antigens that formed the IC, as actMMP-9 did not degrade Igs. The quantification of the peaks is shown in the bar graphs near the gel filtration profiles. The results of these gel filtration chromatography analyses with intact IC in solution corroborated our finding of a function of actMMP-9 in cleaving circulating IC.

### Lack of MMP-9 Correlates With Decreased Clearance of IC *in vivo*

**Figure 6:**
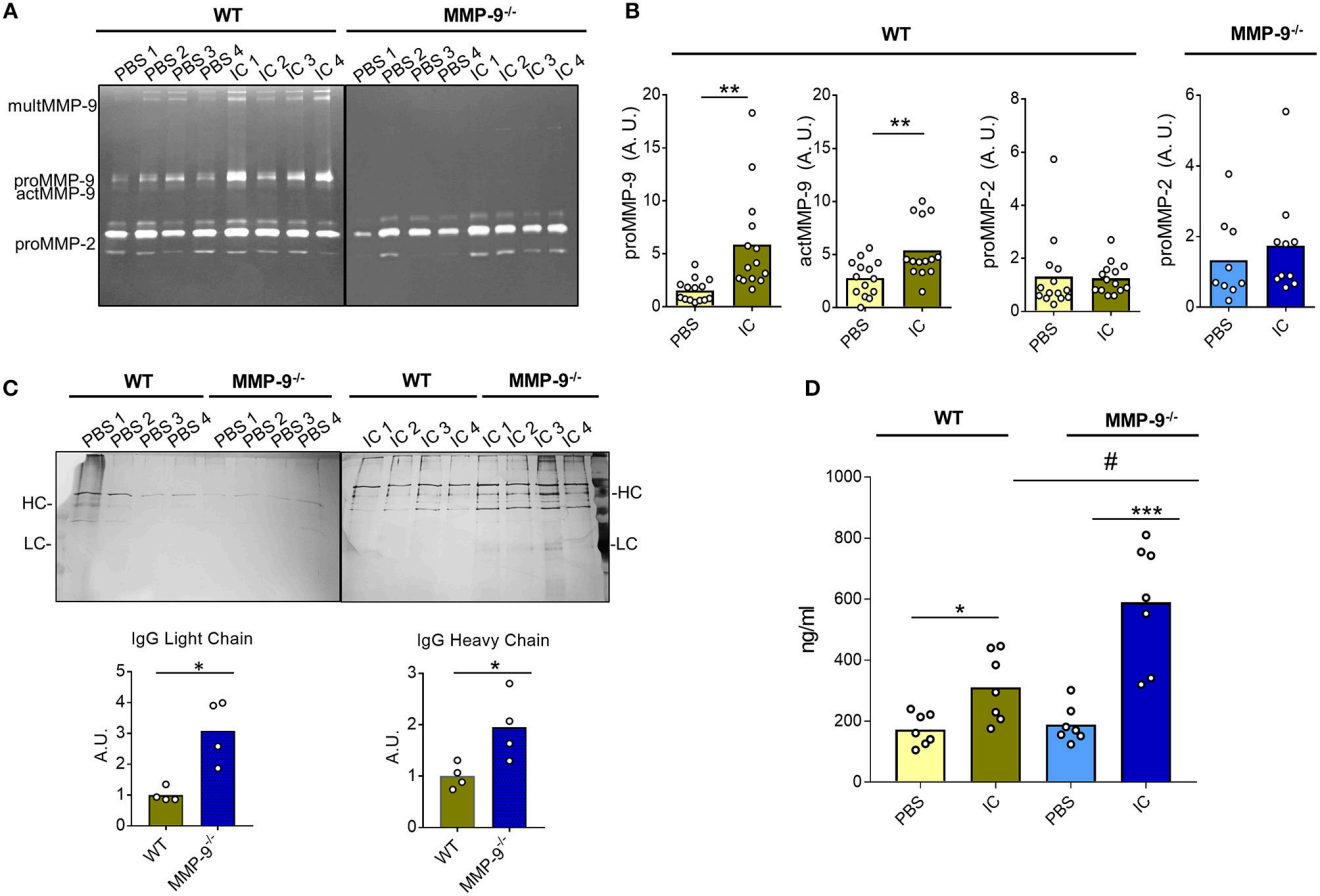
Quantification of IC in air pouches after 24 h. **(A)** Representative gelatin zymography gels from the analysis of the exudates obtained after injection of PBS or IC in the air pouch of WT and MMP-9^−/−^ mice. **(B)** Quantification of the proMMP-9, actMMP-9 and proMMP-2 gelatinolytic signal obtained in the zymographies, as shown in **(A)** from14 WT mice treated with PBS and 14 WT mice treated with IC and 9 MMP-9^−/−^ mice treated with PBS and 10 MMP-9^−/−^ mice treated with IC. Standardization and methodology have been described in detail before (51) **(C)** SDS-PAGE and silver stain analysis of IC proteins from the air pouch after injection of PBS or IC. Prior to analysis, the IC were purified with protein-G-Sepharose. HC, heavy chain; LC, light chain. The graphs underneath the photographs represent the quantification of the heavy and light chains of IgG after injection of IC in the pouch. A.U. = arbitrary units representing the densitometry analysis of the proteins with the use of ImajeJ software. **(D)** ELISA for IgG in the air pouch exudates after injection of PBS or IC in WT or MMP-9^−/−^ mice. *P*-values were determined by ANOVA Kruskal-Wallis test. ^*^p < 0.05, ^**^p < 0.01 and ^***^p < 0.001 comparing PBS and IC conditions in both genotypes and #*p* < 0.05 comparing WT and MMP-9^−/−^ IC conditions. Light yellow histograms represent the data from WT mice injected with PBS, green represents data from WT mice injected with IC, light blue represents MMP-9^−/−^ mice injected with PBS and dark blue represents MMP-9^−/−^ mice injected with IC. Each individual circle represents a data point from a single animal.

As a proof of concept, we investigated the role of MMP-9 in dissolving IC in an *in vivo* model. We used an air pouch animal model, which is a standard test in pharmacological, immunological and biomaterial research (28–30). We compared WT (14 control mice and 14 mice injected with IC) and MMP-9^−/−^ knock out animals (9 control mice and 10 mice injected with IC) for their capacities to dissolve exogenously administered IC. After the air pouch was established, 5 μg of IC were injected and 24 h later the pouch exudates were collected to characterize cells and fluids. First, we analyzed the presence of MMP-2 and MMP-9 by gelatin zymography (Figures 6A,B and Supplemental Figure 15A). We demonstrated significant increases of proMMP-9 and actMMP-9 levels when we injected IC in the WT pouches, whereas the levels of MMP-2 were not altered. As expected, MMP-9 was not present in the MMP-9^−/−^ animals, and, interestingly, MMP-2 levels did not significantly increase in the presence of IC in WT vs. MMP-9^−/−^ mice (Figures 6A,B, Supplemental Figure 15A).

It was important to mention that no differences between WT and MMP-9^−/−^ mice were detected in the amount and types of cells migrating into the pouch after IC or PBS (control) injection (Supplemental Figure 15B). Nevertheless, and as expected, the number of cells increased approximately 10 times after IC injection in both genotypes. No differences were observed in the migration of cells into the pouch between WT and MMP-9^−/−^mice (Supplemental Figure 15B). We also characterized the cell types from the exudates by flow cytometry (Supplemental Figure 15C) and cytospin analysis (Supplemental Figures 15D,E). The characterization of the cells by cytometry revealed that after 24 h neutrophils and mostly macrophages were present in the air pouch when we injected IC in WT and MMP-9^−/−^ mice. The cytometry analysis was corroborated by counting cytospins. Remarkably, the images not only confirmed increased macrophage percentages in the pouch after administration of IC, but also clearly showed the presence of considerable amounts of vacuoles in these cells (red arrows in Supplemental Figure 15E).

Mostly relevantly, we analyzed the presence and alterations of IC in the fluid exudates. We first purified the IC and analyzed these by SDS-PAGE followed by silver staining of proteins. WT and MMP-9^−/−^ controls (injected with PBS) barely contained Ig heavy and light chains in the air pouch exudates, whereas after IC injection, Ig heavy and light chains were detected in both groups (Figure 6C). Significantly, more Ig remained present in the MMP-9^−/−^ than in WT mouse air pouches (Figure 6C). We also examined the amount of remaining IC in the pouches by an anti-IgG ELISA (Figure 6D). The levels of IgG in control animals (PBS treated) were low and similar in both genotypes. As expected, after IC injection slightly increased IgG levels were seen in WT, whereas in MMP-9^−/−^ mice, these increases were significantly more pronounced, suggesting that the absence of MMP-9 delayed the degradation of IC.

Collectively, these data indicated that MMP-9 regulates IC levels not only by degrading aAg and preventing IC formation, but also by degrading the already formed IC.

## Discussion

In the present study, we observed that the levels of IC were significantly higher in the plasma of mice lacking MMP-9, compared with animals with SLE like disease (LPR^−/−^). In the mice lacking MMP-9 only a trend in higher IC accumulation was observed in the spleen and kidneys. This hinted to the fact that the absence of MMP-9 might lead to an increase of IC in plasma and deposition in peripheral organs like kidney, lymph nodes and spleen. We demonstrated that active MMP-9 is capable to degrade IC from plasma samples of SLE patients and from the SLE animal model LPR^−/−^. Interestingly, this clearance role is not by destruction of the immunoglobulins, but instead, and solely, by degradation of the autoantigenic proteins. By analyzing the content of the IC, degraded by MMP-9, we found not only many known MMP-9 substrates, but we also discovered a new autoantigen substrate of MMP-9, the Apolipoprotein B-100. In addition, we showed, *in vivo*, that the lack of MMP-9 delayed the degradation of IC.

**Figure 7:**
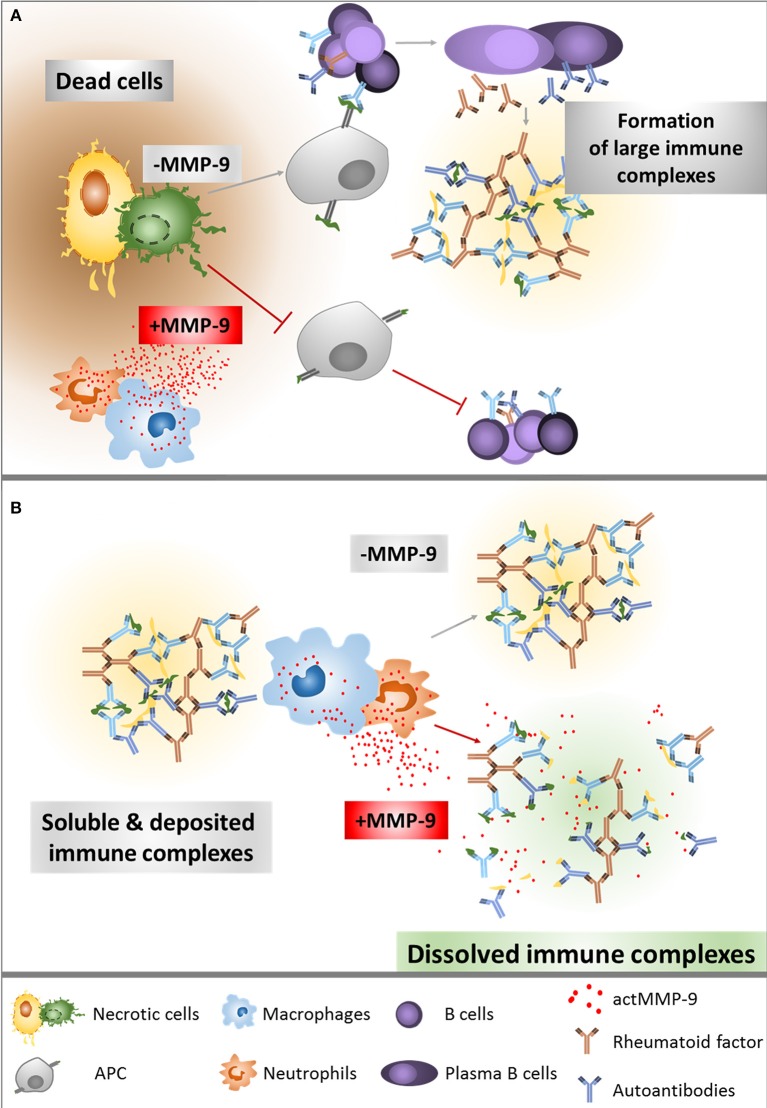
Overview of the role of MMP-9 in the degradation of aAg **(A)** and IC **(B)**. **(A)** After cell necrosis, intracellular proteins are released into the extracellular matrix. In the absence of actMMP-9, these proteins remain intact and highly immunogenic. Classical antigen presentation enhances T helper functions, generating a considerable immune response with antibody secretion. In the presence of actMMP-9 and other proteases, the intracellular proteins are cleaved, diminishing the immunogenic capacity and therefore generating a lower or no immune response. **(B)** Once the IC are formed, actMMP-9 may be released by neutrophils and macrophages. As a result, actMMP-9 degrades the antigens within the complexes, making the IC smaller and therefore easier to be phagocytosed and cleared by macrophages and neutrophils. The lack of actMMP-9 helps to preserve the large IC in intact form and, as a consequence, the deposition of IC and tissue damage are increased.

Cauwe et al. already titrated the levels of some aAb against established MMP-9 substrates, for instance anti-actin or anti-tubulin. The serum concentrations of these aAb were significantly increased in the LPR^−/−^ lupus mouse model that lacks MMP-9, compared with the single LPR^−/−^ mice (23). This finding suggested that MMP-9 clears aAg released from apoptotic and necrotic cells (Figure 7A). Interestingly, aAb against non-MMP-9-substrates, such as anti-Smith IgG, anti-Histone IgG or anti-Chromatin IgG and rheumatoid factor IgG were also increased in the double knockout LPR^−/−^/MMP-9^−/−^ (23). This suggests that MMP-9 has an additional protective role in SLE, besides the autoantigen degradation. Degradation and clearance of IC will prevent its deposition in several organs and avoid tissue damage. Follicular dendritic cells in the spleen are involved in immune complex trapping and help to clear the IC from the blood with the help of macrophages (24, 31). Therefore, after observing that IC levels were higher in MMP-9^−/−^ mice, we hypothesized that MMP-9 might disaggregate large IC already deposited in the spleen and also in the peripheral blood circulation to facilitate their phagocytosis and degradation by macrophages (Figure 7B).

It is established that MMPs are highly regulated proteases that degrade substrates *in vivo* under well-defined conditions. We showed that neither MMP-9 nor MMP-1,-2,-3 and -8 cleave immunoglobulins IgM and IgG. This is a surprising finding in view of the fact that immunoglobulins are proteins of considerable sizes. Both, immunoglobulins (e.g., IgG and IgM) and MMPs (e.g., MMP-9) mediate critical immune functions and may co-exist in the blood stream and tissues, thus acting together. Our data support the important role of MMPs in the immune system by cleaving antigens while leaving IgG and IgM intact. For comparison, typical proteases in the gut, like trypsin and chymotrypsin, efficiently digest IgG (32).

We showed that actMMP-9 cleaves known aAg, such as actin and αB-crystallin within the context of IC. The nature of the antigen and the used antibody preparations influenced the degree of autoantigen protection against proteolysis suggesting that the *in vivo* half-lives of IC with protein aAg might vary considerably.

Regarding the IC used to study the cleavage by actMMP-9, we were aware that in our preparations, protein G only selected for IC formed by IgG and that IC based on IgM thus were excluded. However, for the purpose of the present work, it was more critical to obtain pure IC, not contaminated with other plasma proteins, than to collect all lupus-related IC of all possible Ig (sub)classes.

The degradation of the IC was studied in human SLE plasma samples and, similarly, in plasma samples obtained from two SLE mouse lines (LPR^−/−^ and LPR^−/−^/MMP-9^−/−^), with varying degrees of SLE-like phenotypes. Both, human and murine IC samples were degraded by actMMP-9, although the switches in the banding patterns varied depending on the plasma samples, in line with the heterogeneity of IC between SLE patients (33). The protein identification of plasma IC proteins degraded after incubation with actMMP-9 provided interesting information. Firstly, most of the identified proteins were already known substrates of MMP-9. This was the case for C4 (34), fibrinogen (35), fibronectin (36), and C1q (37). All proteins identified here as substrates of MMP-9 are also autoantigens in SLE (12). Consequently, autoantibodies against C4 (38), fibrinogen (39), fibronectin (40), and C1q (41) have been described in SLE. From our search of MMP-9 substrates within IC, we serendipitously discovered a new substrate of MMP-9, namely Apo-B 100. In line with our hypothesis (Figure 7B), autoantibodies against Apo-B 100 have been described in SLE plasma samples (42). After incubation with actMMP-9, the immunoreactive signal of Apo-B 100 disappeared, rather than yielding immunoreactive fragments, suggesting that MMP-9 cleaves at several sites within this protein. Although further biochemical analysis is needed to define the MMP-9 cleavage sites, the protease specificity prediction server “PROSPER” provided preliminary information about the Apo-B 100 cleavage sites generated by actMMP-9. With PROSPER analysis we found that actMMP-9 may cleave at more than 250 sites within the human Apo-B 100 amino acid sequence.

Finally, we used the mouse air-pouch model to prove, *in vivo*, the role of MMP-9 in IC clearance. We found that the levels of proMMP-9 and actMMP-9, but not of MMP-2, were significantly increased in the WT animals after administration of IC. After IC injection in the pouch, 10 times more cells and a strong shift in the proportion of monocytes and macrophages were observed in both WT and MMP-9^−/−^ mice, whereas the IC clearance analyzed by ELISA and SDS-PAGE quantification was significantly higher in WT vs. MMP-9^−/−^ mice. It is known that macrophages are able to clear IC, and that SLE patients have a deficiency in IC clearance due to macrophage and complement system defects (43). M2 macrophages are stimulated by IC and consequently are more implicated in IC clearance than M1 macrophages (44). Remarkably, M2 macrophages produce more MMP-9 and less TIMP-1 (45). These data support a role for macrophage MMP-9 activity in IC clearance.

Several compounds have been suggested to cause drug-induced lupus erythematosus (DILE): hydralazine, procainamide, quinidine, doxycycline, isoniazid, diltiazem, minocycline, and D-penicillamine (6, 7). Interestingly, some of these drugs are known to inhibit MMP-9. For example, doxycycline, described as DILE inducer (8), has been used as MMP-9 inhibitor in lung injury (46) and aortic valve disease induced by cardiopulmonary bypass (47). Similarly, D-penicillamine has also been described to induce lupus (48) and is an MMP-9 inhibitor, known to delay experimental autoimmune encephalomyelitis (49). An inevitable consequence is that, before using small- or broad-spectrum MMP-9 inhibitors in SLE patients, careful preclinical evidence of efficiency needs to be provided and sufficient safety measures will be needed in clinical trials. Based on this information about DILE and our new data, we suggest that MMP-9 acts as a major beneficial factor in SLE by clearing aAg and IC.

We provided here biochemical, immunological and biological as well as preclinical and clinical data that demonstrate a beneficial role of MMP-9 in SLE, not only by cleaving aAg, but also by clearance of IC. In this way, MMP-9 complements the complement system in IC clearance. Finally, we believe that the value of the present work is relevant for other (systemic) autoimmune diseases in which IC are pathogenic.

## Materials and Methods

### Mice

The generation of MMP-9 deficient mice was described previously (50). LPR^−/−^ mice on a C57Bl/6 background were obtained from the Jackson Laboratory (Bar Harbor, ME, USA). To generate LPR^−/−^MMP-9^−/−^ knock out mice, we crossed MMP-9^−/−^ and LPR^−/−^ mice. F1 heterozygote animals were mated to obtain LPR^−/−^MMP-9^−/−^ in the F2 generation. The genotype of every mouse in the present study was defined by PCR. We used in all forthcoming experiments black animals from the 13th generation backcross into C57Bl/6. All mice were bred in specific pathogen-free (spf) insulators at the Rega Institute for Medical Research and moved to non-SPF conditions after weaning. All experimental procedures were approved by the institutional Ethics Committee under license LA1210243 for animal welfare (Project 277/2014). The numbers of female and male animals were similar and the diseases scores between both sexes were not different, as was already described for this SLE mouse model (22, 23). Late disease stage samples of mice were collected between months 7 and 9, when the SLE disease was detectable. As was described before, a high variability exists between the disease scores within specific genotypes (22, 23).

### Immunohistochemistry Analysis

Paraffin-embedded spleens and lymph nodes (data not shown) were sliced into 5 μm sections and dried overnight at 50°C. For immunohistochemical staining the EnVisionTM FLEX kit (DAKO) was used. Goat anti-mouse C3d (R&D Systems) and isotype control were used as primary antibody and peroxidase-labeled secondary antibodies as detection system. Quantification analysis was done with the “Fiji” version of ImageJ, by measuring the mean intensity of the signal of DAB (deconvolution of the colors: color 2 = DAB) and converting it to Optical Density (OD) by the formula. OD = log(max intensity/Mean intensity), where max intensity = 255 for 8-bit images.

### Spleen and Kidney Tissue Extractions

Mice were sacrificed and organs were collected. Halve spleens and complete kidneys from individual mice were homogenized in hard tissue homogenizing CK28 tubes (Bertin Technologies) in 0.5 ml of assay buffer (50 mM Tris pH 7.4, 150 mM NaCl, 5 mM CaCl_2_, 0.01% Tween-20) and proteins were extracted with a Precellys lysing kit (Bertin Technologies) using the Precellys® 24 (2 times 5 s at 6,000 g, Bertin Technologies). To precipitate all tissue debris, the samples were centrifuged at 20,000 g and 4°C for 15 min. The supernatant, containing soluble proteins, was collected.

### ELISAs

A specific sandwich ELISA for C3d in tissue extracts from mice was developed by using the combination of a specific polyclonal Ab against mouse C3d as the coating Ab (Goat anti mouse C3d, art. AF2655 from R&D Systems; coating concentration 4 μg/ml in 0.1 M NaHCO_3_ pH 9.6) and a monoclonal detection Ab against mouse C3 with affinity for the C3d fragment (Rat mAb to mouse C3 art. ab11862 from Abcam; conc 0.2 μg/ml in PBS with 0.5% casein and 0.05% Tween 20). The detection was done with the use of a HRP conjugated anti Rat Ab (art.112-035-143 from Jackson ImmunoResearch Laboratories, 0.1 μg/ml) and the peroxide TMB staining system. For anti IgG ELISAs, 96 well plates were coated with 2 μg of anti-human IgG antibody. After washing and blocking the plates, the studied samples were added and incubated overnight at 4°C. The plates were then washed and incubated with specific secondary HRP-conjugated antibodies against human IgG for 1 h at RT.

### Gel Filtration Chromatography and Protein Quantification Analysis

Samples were loaded on a Superdex 200 HR 10/300 column (GE Healthcare Life Sciences) equilibrated with 20 mM sodium phosphate pH 7.4; 150 mM NaCl at 0.4 ml/min flow rate and 0.5 ml fractions were collected. The absorption peaks at 280 nm obtained from 2.5 μl samples after gel filtration chromatography were analyzed with the Unicorn 5.1 software (GeHealthcare Life Sciences). Peak integration was used to measure peak areas. The relative areas of the main peaks were calculated against the total areas.

### SDS-PAGE, Protein Staining and Gelatin Zymography Analyses

Samples were used in native form or chemically reduced and buffered and proteins were separated on Tris-glycine gels (Invitrogen, Carlsbad, CA, USA). Next, proteins were stained for further processing by Coomassie Brilliant Blue staining or with the SilverQuest™ Silver Staining Kit (Invitrogen, Carlsbad, CA, USA). Gelatin zymography gels were prepared and consisted of a 7.5% acrylamide separating gel with 1 mg/ml gelatin (Sigma Aldrich G1890), topped with a 5% stacking gel. After electrophoretic protein separation, the gels were washed twice for 20 min in re-activation solution (2.5% Triton-X-100). Afterwards, the gel was incubated overnight in 10 mM CaCl_2_ and 50 mM Tris–HCl, pH 7.5 at 37°C. Staining was performed with 0.1% Coomassie Brilliant Blue R-350 (GE Healthcare) and zymograms were analyzed densitometrically using the ImageQuant TL software (GE Healthcare) (51).

### *In vitro* Cleavage of Antibodies and aAg by MMP-9

IgGs (2 μg) were incubated with active MMP-9 at 37°C at a ratio actMMP-9:IgG of 1:5 or 1:50. After 6 or 24 h, the samples were analyzed by SDS-PAGE and protein staining with Coomassie blue. EDTA (MMP inhibitor) and actMMP-3 (for activation of proMMP-9) were used as controls. For the study of other MMPs, active MMP-1,−2,−3,−8 and also MMP-9 were incubated at a ratio enzyme: Ig of 1:5 for 24 h and the results of the incubations were analyzed by SDS-PAGE and Coomassie blue staining of proteins. To prepare the IC with actin or αB-crystallin (as substrates for MMP-9), 1 μg of the proteins were incubated with their respective antibodies at a ratio Ab:substrate 2:1. Afterwards, the IC were incubated with actMMP-9 at a ratio enzyme substrate 1:20. The resulting products of the incubations were visualized after SDS-PAGE by silver staining.

### Human Plasma Samples

Blood samples from SLE patients were centrifuged at RT and 1,500 rpm for 5 min. The resulting supernatants were used as plasma samples. All donors gave written consent and all procedures were according to the terms of the declaration of Helsinki and following Belgian and European legislation (Ethical committee reference number: S58110). In Supplemental Table 1 information about the SLE patients is provided. All patients suffered from clinical SLE symptoms and were under various anti-inflammatory treatments at plasma sampling.

### Purification of IC

IC and large and anionic proteins were precipitated by dilution of 200 and 100 μl of human and mouse plasma, respectively, with an equal volume of 7% of PEG 6000 at 4°C overnight. After incubation, the samples were centrifuged at 13,000 rpm and washed 3 times with 3% PEG 6000. Subsequently, the precipitated material was dissolved in 200 or 100 μl phosphate buffered saline (PBS) for human and mouse samples, respectively. The IC were then purified by precipitation with protein-G-Sepharose beads, washed and eluted with 0.1 mM glycine at pH 2.2. The pH was adjusted to 7 with Tris buffer.

### Cleavage of IC by MMP-9 and Densitometry Quantification

IC purified from mouse and human plasma samples were incubated with actMMP-9 (final concentration 0.15 μM) during 16 h at 37°C. The products of the incubation were separated by SDS-PAGE and visualized by silver staining. For quantification of the different protein band patterns we used ImageJ software.

### nanoLC/TOF/MS and Protein Identification

The nano liquid chromatography/Time-of-flight/ Mass spectrometry (nanoLC/TOF/MS) and protein identification were done by Alphalyse (Alphalyse A/S, 5220 Odense, Denmark). Briefly, the protein samples were reduced and alkylated with iodoacetamide, i.e., carbamidomethylated, and subsequently digested with trypsin. The resulting peptides were concentrated by Speed Vac lyophilization and redissolved for injection on a Dionex nano-LC system and MS/MS analysis on a Bruker Maxis Impact QTOF instrument. The MS/MS spectra were used for Mascot database searching. The data were searched against in-house-Alphalyse protein databases downloaded from UniProt and NCBI containing more than 80 million known non-redundant protein sequences. The Mascot software found all the matching proteins in the database by their peptide masses and peptide fragment masses. The protein identification was based on a probability-scoring algorithm (www.matrixscience.com). It was considered a positive identification when at least 2 peptides had an MS-ions score above 35 or if a protein under 20 kDa had 1 peptide with an MS-ions score above 50.

### Air Pouch Assay

The air pouch analysis was performed as detailed previously (30). Briefly, dermal air pouches were generated by injecting mice at dorsal sites with 3 ml of filtered air on days 0 and 3. The negative control sample consisted of phosphate-buffered saline (PBS). On day 6 the IC and control preparations (PBS) were injected. After 24 h, the exudates of the pouches were collected in 5 ml of PBS to characterize the viable cells by cytospin and flow cytometry analysis and the supernatant fluids for gelatin zymography and IC detection.

### Cytospin Analysis

75 × 10^3^ cells were applied onto slides by centrifugation using a Shandon Cytospin 2 apparatus (Thermo Shandon, Pittsburgh, USA). Then the cells were stained with Hema-color (Merck Chemicals, Darmstadt, Germany). Hundred cells were counted and classified based on morphology and staining pattern. The average of 3 counts per slide was represented.

### Flow Cytometry Analysis

To corroborate cytospin analysis, we also performed flow cytometry analysis. 10^6^ cells were incubated with Fc-receptor-blocking antibodies (anti-CD16/anti-CD32, Miltenyi Biotec), and with the Zombie Aqua Fixable Viability Dye. Fifteen minutes later, the cells were washed with PBS+2% FCS and then stained for 30 min with the following antibodies: Gr-1, Ly6G, Ly6C, CD11b, CD11c, F4/80, (eBioscience, BD Biosciences or BioLegend). After incubation, the cells were washed and fixed with 0.37% formaldehyde. Cells were analyzed on a fluorescence-activated cell sorting (FACS) Fortessa X20 (Becton Dickinson, San Jose, CA, USA), excluding dead cells by the Zombie Aqua Fixable Viability Dye (BioLegend). Live singlet cells were analyzed with FlowJo (Version 10).

## Statistical Analyses

Statistical analysis were performed using GraphPad Prism 6 software. Significant differences between experiments were evaluated using a non-parametric ANOVA Kruskal-Wallis test. All *p*-values of 0.05 or less were considered significant.

## Ethics Statement

This study was carried out in accordance with the recommendations of the Belgian and European legislation, Ethical committee reference number S58110. All subjects gave written informed consent in accordance with the Declaration of Helsinki. All mouse experimental procedures were approved by the institutional Ethics Committee under license LA1210243 for animal welfare (Project 277/2014).

## Author Contributions

EU-B and GO designed the study. EU-B designed the experiments and conducted most of the research, EM, LB, JV, VR, DB, PP executed specific experiments, EU-B and GO wrote the manuscript with input from all authors.

### Conflict of Interest Statement

The authors declare that the research was conducted in the absence of any commercial or financial relationships that could be construed as a potential conflict of interest.

## Supplementary Material

Supplemental Figure 1–8 Immunohistochemistry analysis (IHC) for C3d of the spleens of 2 WT mice (Supplemental Figures 1, 2), 2 MMP-9^−/−^mice (Supplemental Figures 3, 4), 2 LPR^−/−^ mice (Supplemental Figures 5, 6) and 2 LPR^−/−^/MMP-9^−/−^mice (Supplemental Figures 7, 8). Two pictures at three different magnifications (10x, 20x, and 40x) were shown for each mouse. The horizontal bars indicate 50 μm. The quantification of the signal from C3d of these IHC pictures has been used for the graph shown in Figure 1B.

Supplemental Figure 9 IHC analysis of the spleens from the four different genotypes WT, MMP-9^−/−^, LPR^−/−^ and LPR^−/−^/MMP-9^−/−^ with an isotype control antibody (anti-goat).

Supplemental Figure 10 Immunohistochemistry analysis (IHC) for C3d of the kidneys of 2 WT mice, 2 MMP-9^−/−^mice, 2 LPR^−/−^ mice and 2 LPR^−/−^/MMP-9^−/−^mice.

Supplemental Figure 11 Complete set of primary data of purified IC from 8 WT, 8 MMP-9^−/−^, 10 LPR^−/−^, and 10 LPR^−/−^/MMP-9^−/−^ mice, analyzed by SDS-PAGE and Commassie blue staining. The quantification of the heavy chains of these IC has been used to generate the graph shown in Figure 2B. The top left panel is the same as Figure 2A.

Supplemental Figure 12 Gel filtration analysis profiles of plasma samples from 5 WT (yellow), 5 MMP-9^−/−^ (blue), 5 LPR^−/−^ (red), and 5 LPR^−/−^/MMP-9^−/−^ (green) mice, used to generate the averaged graph shown in Figure 2D.

Supplemental Figure 13 Comparisons of gel filtration chromatography profiles from plasma samples at 3 and 9 months for the 4 mouse genotypes. Protein amounts in solutions were determined by absorbance analysis at 280 nm. Representative sequential profiles of one animal for each genotype are provided.

Supplemental Figure 14 Antibodies IgG and IgM and IC of αB-crystallin and actin incubated with actMMP-9. Red arrows indicate the intact autoantigens and substrates of MMP-9. IgG **(A)** or IgM **(B)** immunoglobulins were incubated with the indicated active MMPs during 24 h. The proteins in the SDS-PAGE gels were stained with Coomassie Blue. HC, Heavy chain; LC, Light chain. Actin **(C)** or αB-crystallin **(D)** in free form or within IC with polyclonal (pAb) or monoclonal antibody (mAb) incubated with actMMP-9 at 37°C. After 24 h the proteins were separated by SDS-PAGE and analyzed by silver staining. **(E)** Free αB-crystallin (red arrow) or in a pAb-IC incubated with actMMP-9 for 1, 8, or 24 h at 37°C. After incubation, SDS-PAGE separation and silver staining analysis of the proteins were performed.

Supplemental Figure 15 Gelatin zymography gel analysis of the exudates obtained after injection of PBS or IC in the air pouch of WT and MMP-9^−/−^ mice and analysis of the cells migrated into the air pouch after PBS or IC injection. **(A)** Quantification of the bands of proMMP-9, actMMP-9, and MMP-2 was included to generate the graphs shown in Figure 6B. **(B)** Histograms representing theabsolute numbers of cells collected in the air pouch experiment. **(C)** Flow cytometry analysis of relative cytospin counts of the lavage exudates from the air pouch after injection of PBS or IC in WT and MMP-9^−/−^ mice. For flow cytometry analysis, monocytes were defined by CD11b and Gr-1, macrophages by CD11b and F4/80, neutrophils by CD11b and Ly6G and dendritic cells by CD11b and CD11c as surface markers. **(D)** Cytospins were stained with hemacolor, the cells were identified on the basis of their morphology and the relative cell percentages provided as cumulative histograms. Donut cells represent neutrophils with donut-shaped nuclei. The discrimination between monocytes and macrophages was made on the basis of size and presence of vacuoles. **(E)** Representative images from cytospins of the air pouch lavages after PBS and IC injection in WT and MMP-9^−/−^ mice.

Supplemental Table 1 Information of the SLE patient cohort of the study. The individual patient numbers (P1-P10) refer to the numbers used throughout the manuscript.

Supplemental Table 2 Proteins identified by nanoLC-MS/MS peptide sequencing and database search. The numbers (Band 1, 2, 3, and 4) refer to the excised gel slices within the red rectangles in Figure 5C.
