# Supplementary figures and images for: MMP-9/Gelatinase B Degrades Immune Complexes in Systemic Lupus Erythematosus
Source: Front Immunol. 2019 Mar 22;10:538. doi: 10.3389/fimmu.2019.00538 (PMC6440319; doi:10.3389/fimmu.2019.00538)

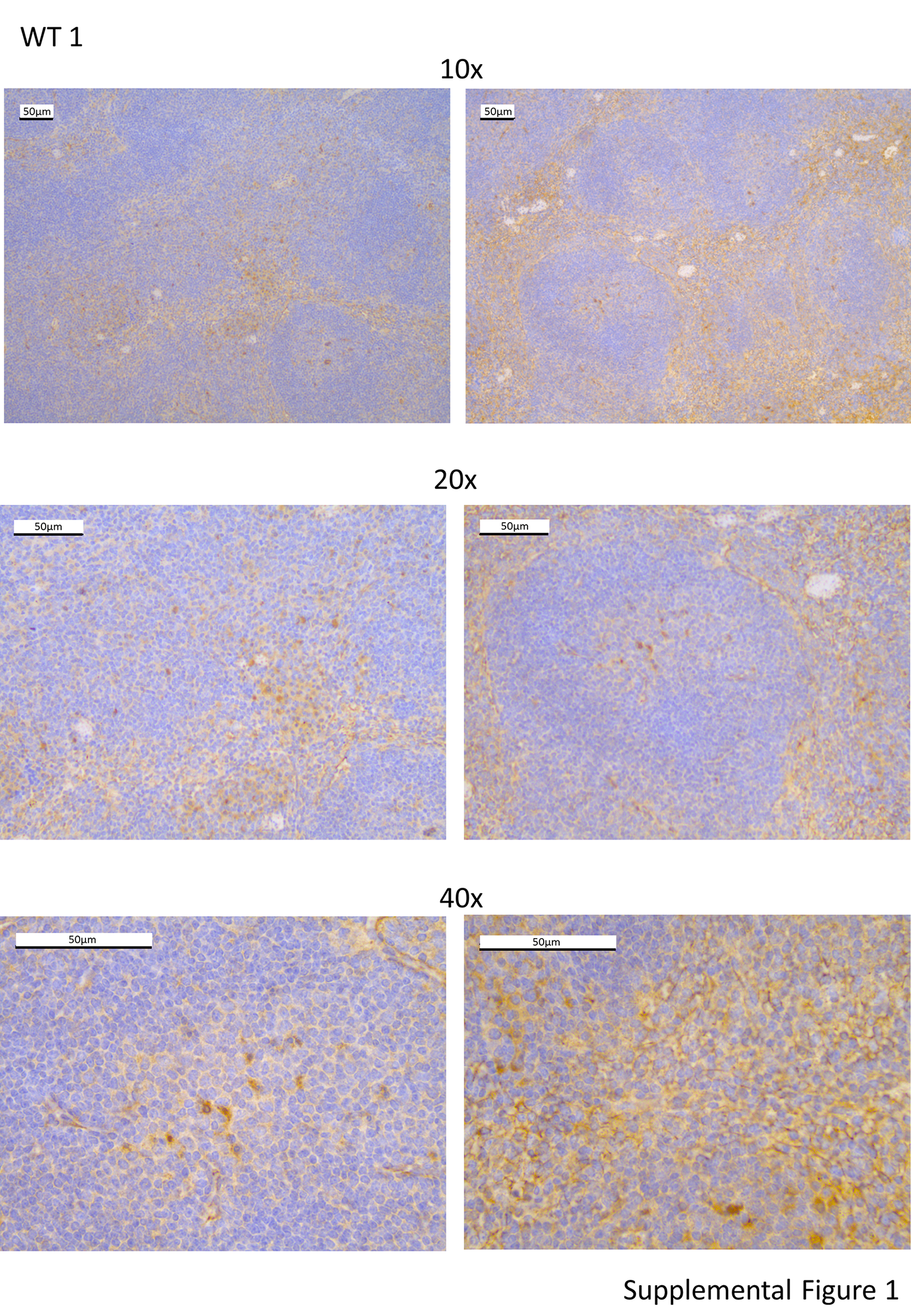

Supplement: Supplemental Figure 1–8 — Immunohistochemistry analysis (IHC) for C3d of the spleens of 2 WT mice (Supplemental Figures 1, 2), 2 MMP-9−/−mice (Supplemental Figures 3, 4), 2 LPR−/− mice (Supplemental Figures 5, 6) and 2 LPR−/−/MMP-9−/−mice (Supplemental Figures 7, 8). Two pictures at three different magnifications (10x, 20x, and 40x) were shown for each mouse. The horizontal bars indicate 50 μm. The quantification of the signal from C3d of these IHC pictures has been used for the graph shown in Figure 1B. [file Image_1.TIF]

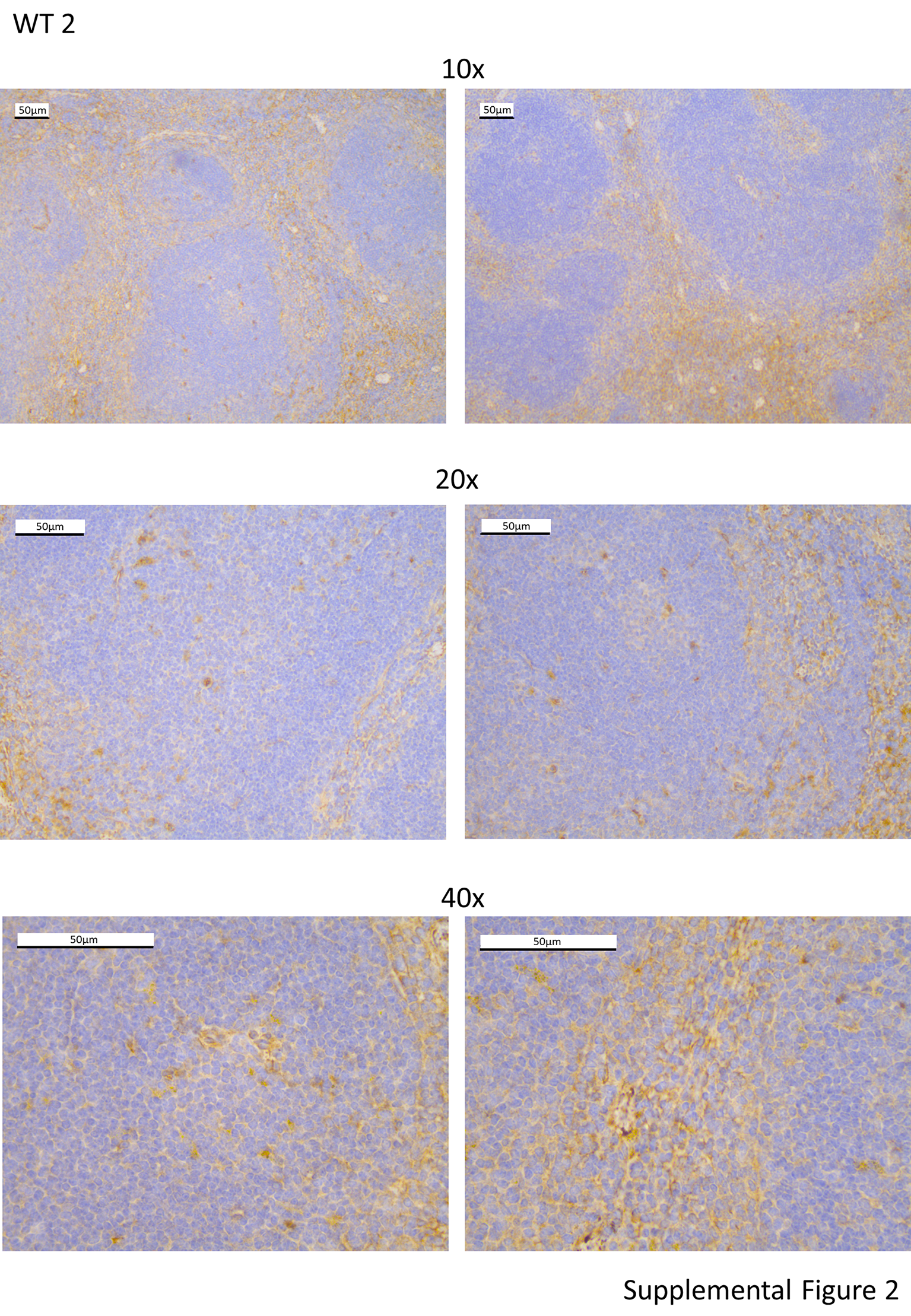

Supplement: Supplementary file 2 [file Image_2.TIF]

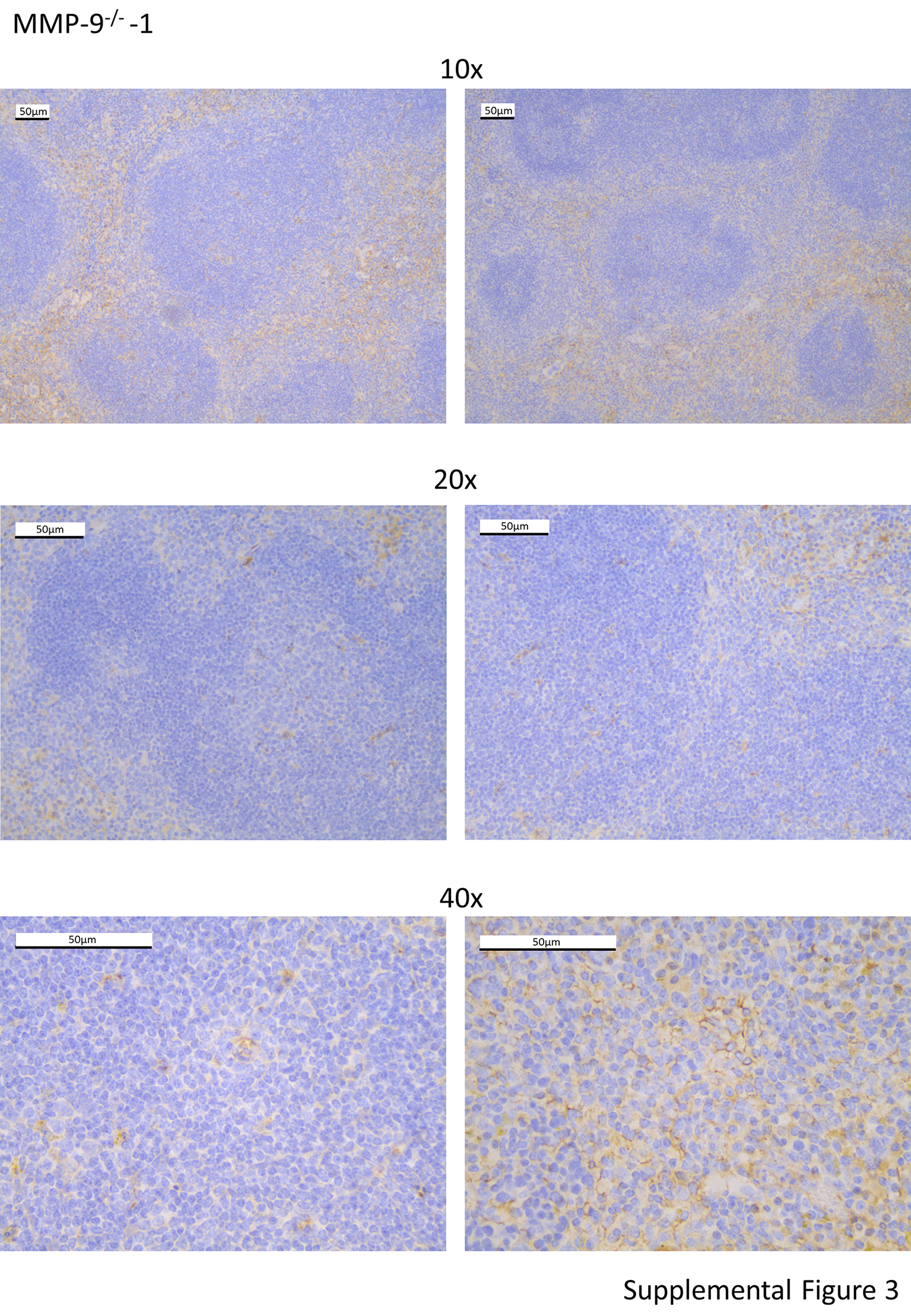

Supplement: Supplementary file 3 [file Image_3.TIF]

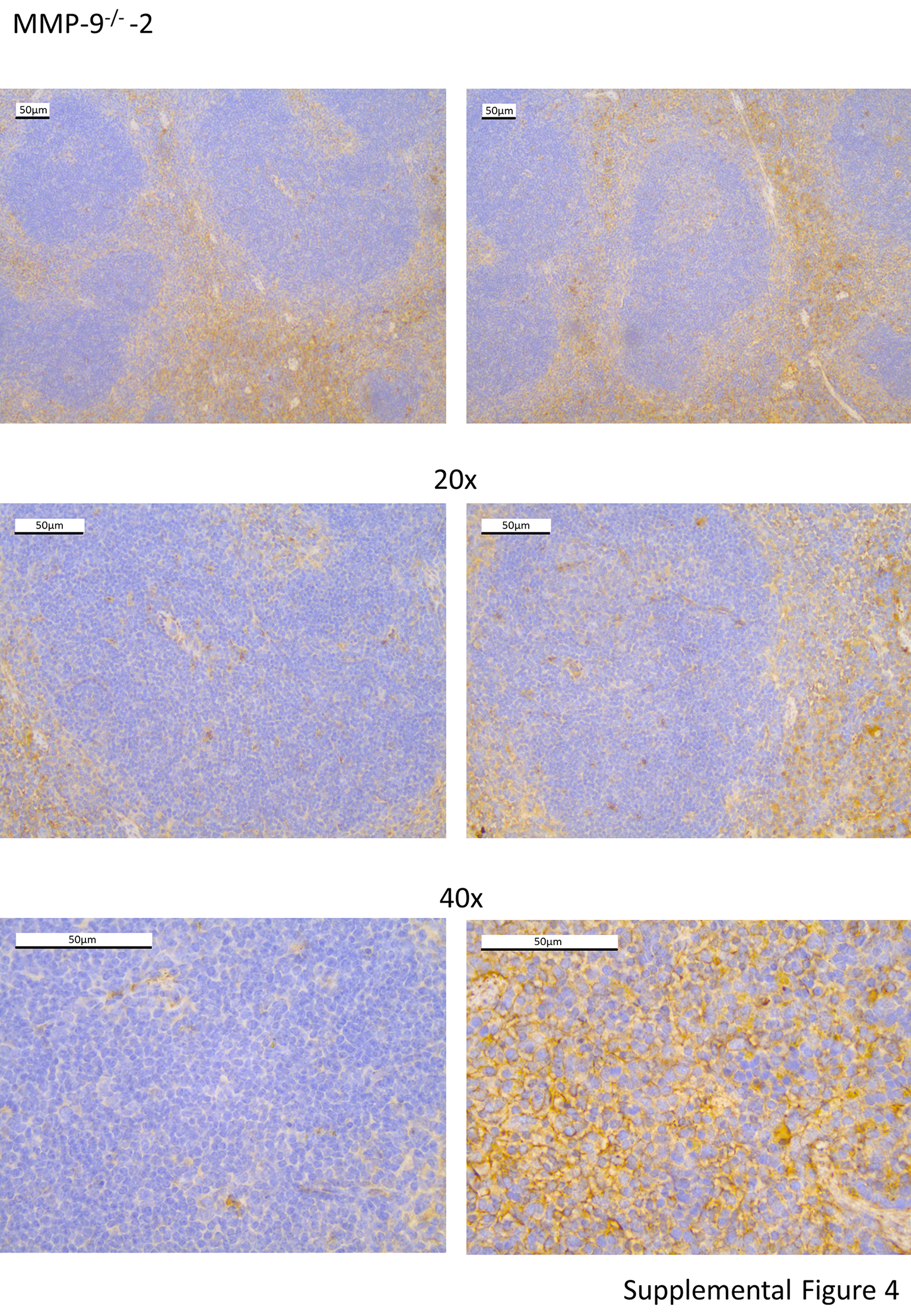

Supplement: Supplementary file 4 [file Image_4.TIF]

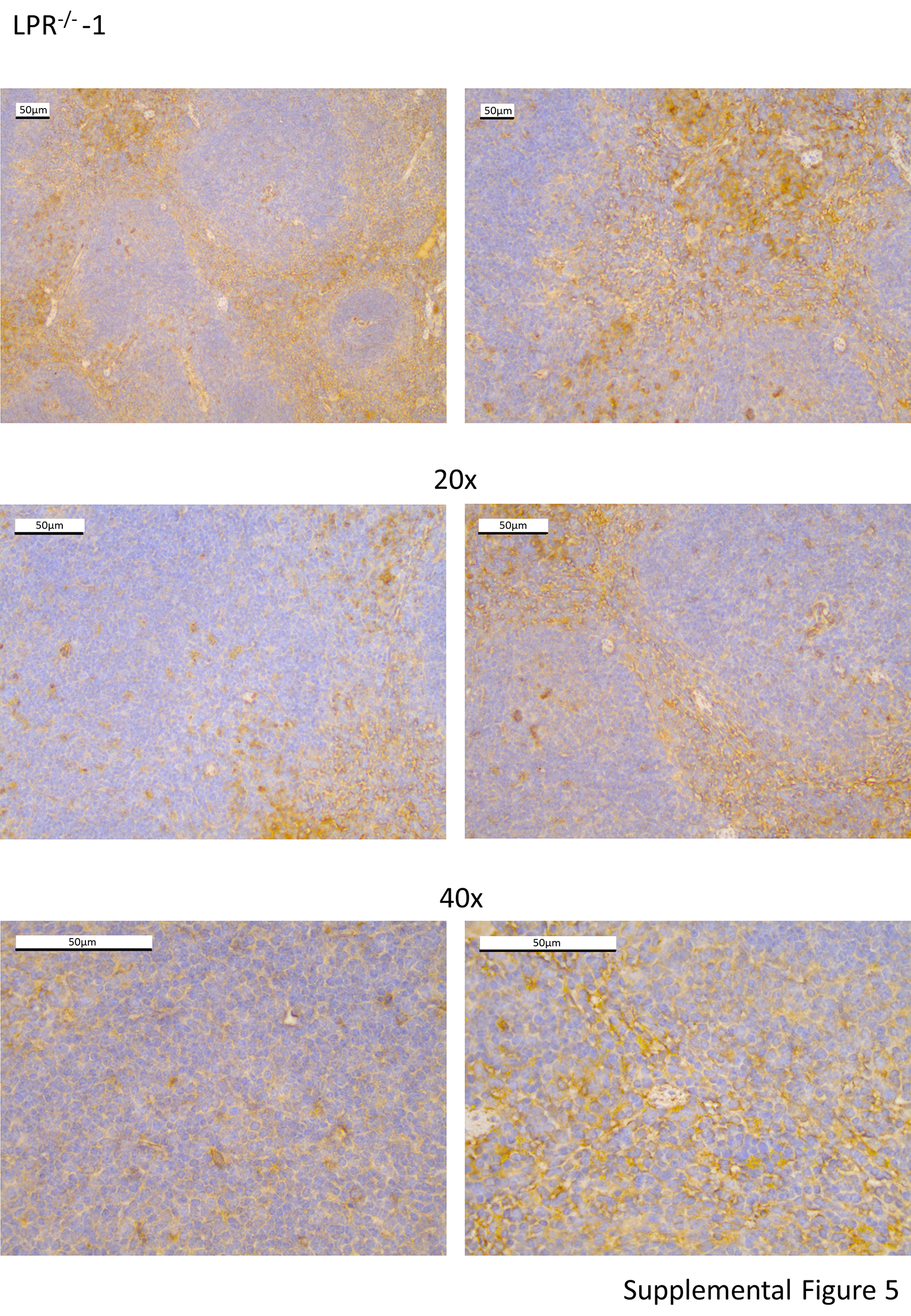

Supplement: Supplementary file 5 [file Image_5.TIF]

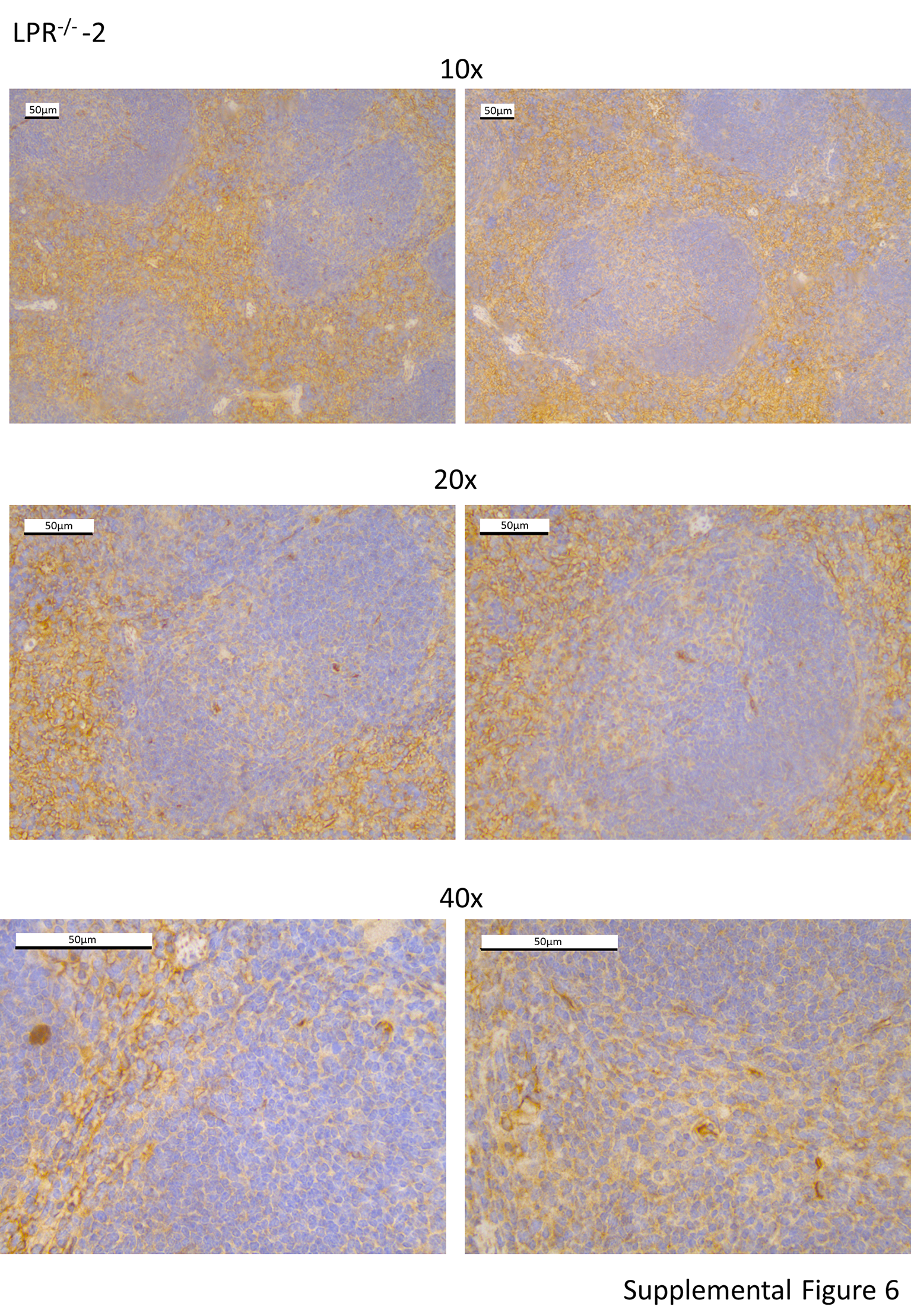

Supplement: Supplementary file 6 [file Image_6.TIF]

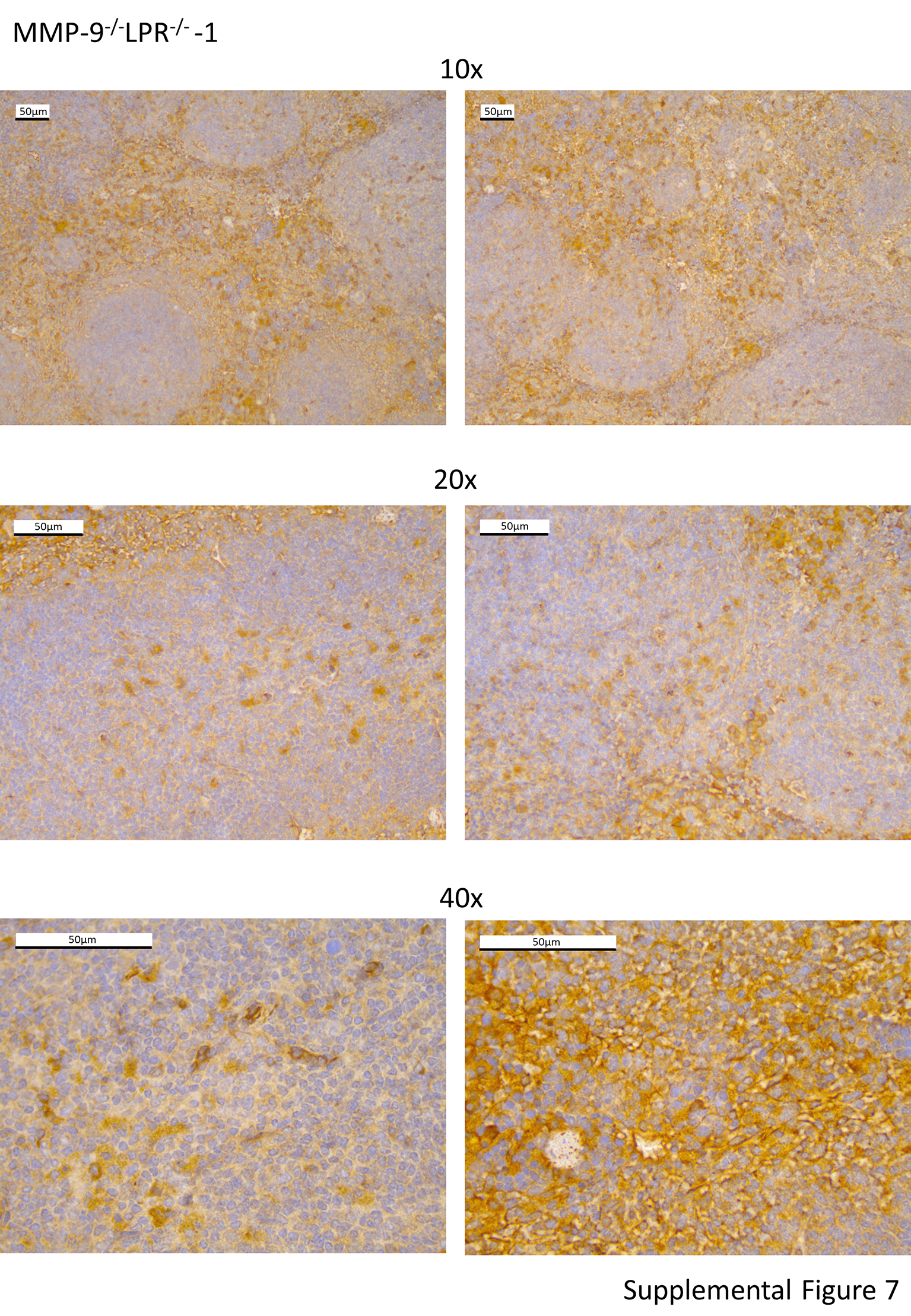

Supplement: Supplementary file 7 [file Image_7.TIF]

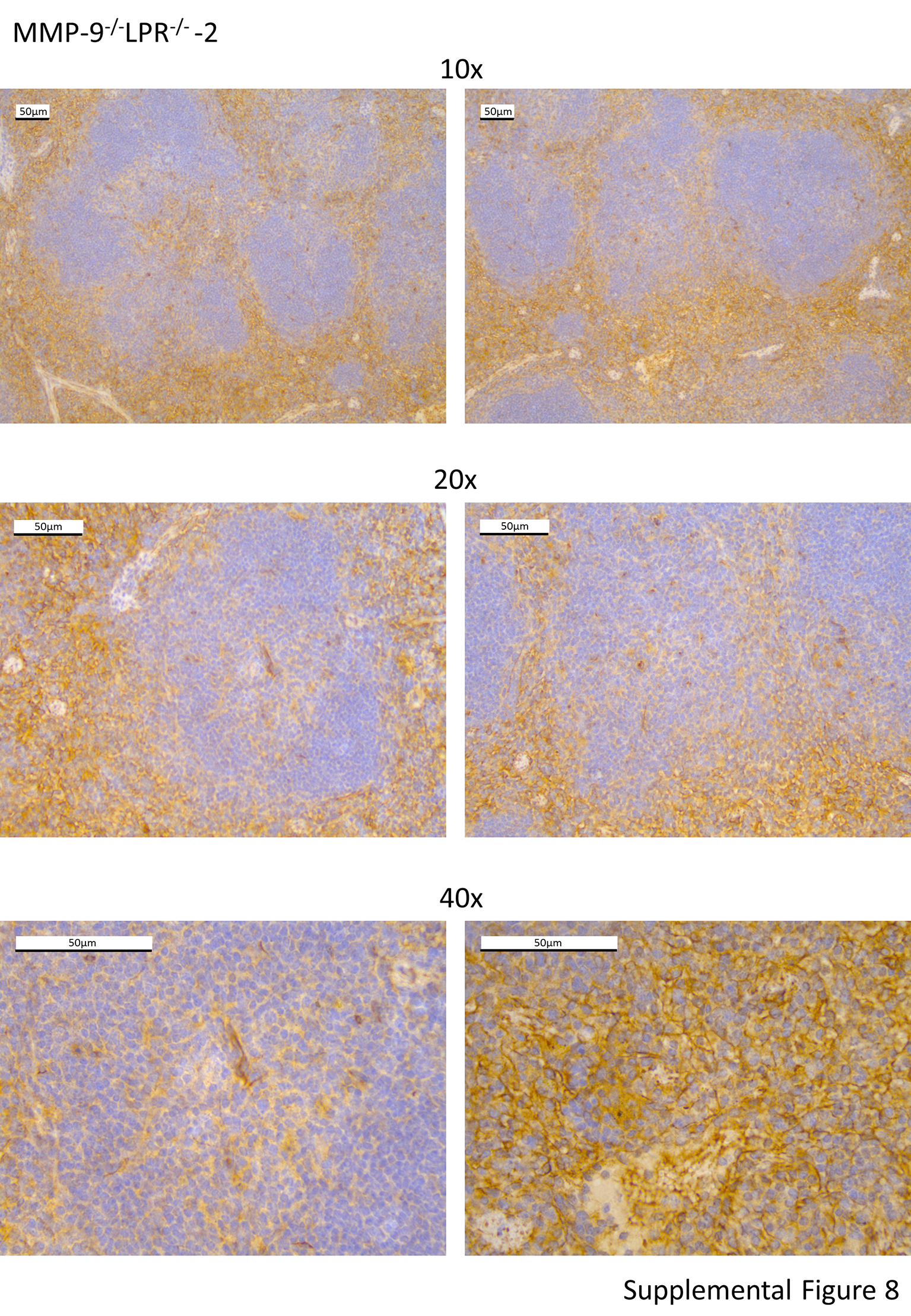

Supplement: Supplementary file 8 [file Image_8.TIF]

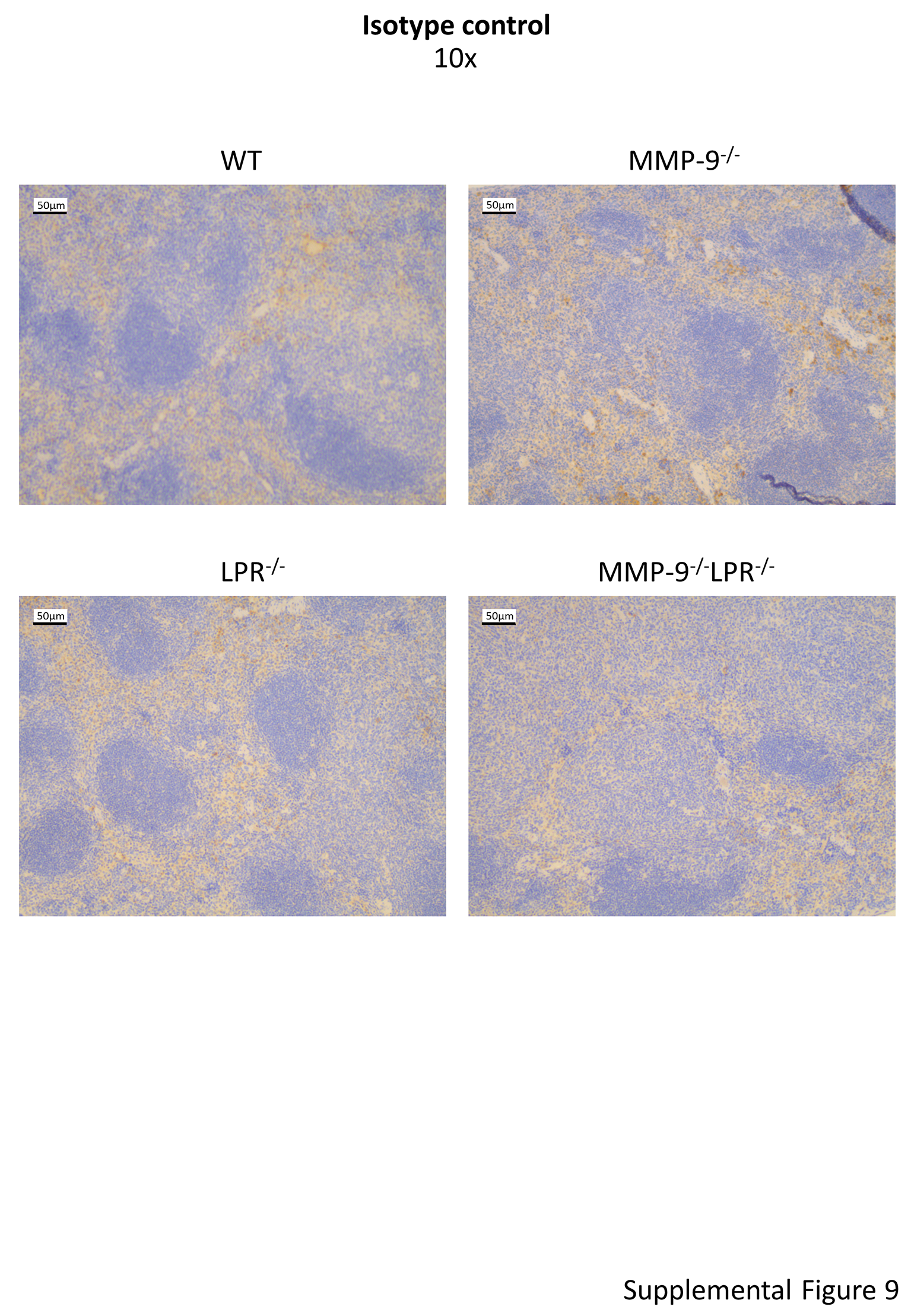

Supplement: Supplemental Figure 9 — IHC analysis of the spleens from the four different genotypes WT, MMP-9−/−, LPR−/− and LPR−/−/MMP-9−/− with an isotype control antibody (anti-goat). [file Image_9.TIF]

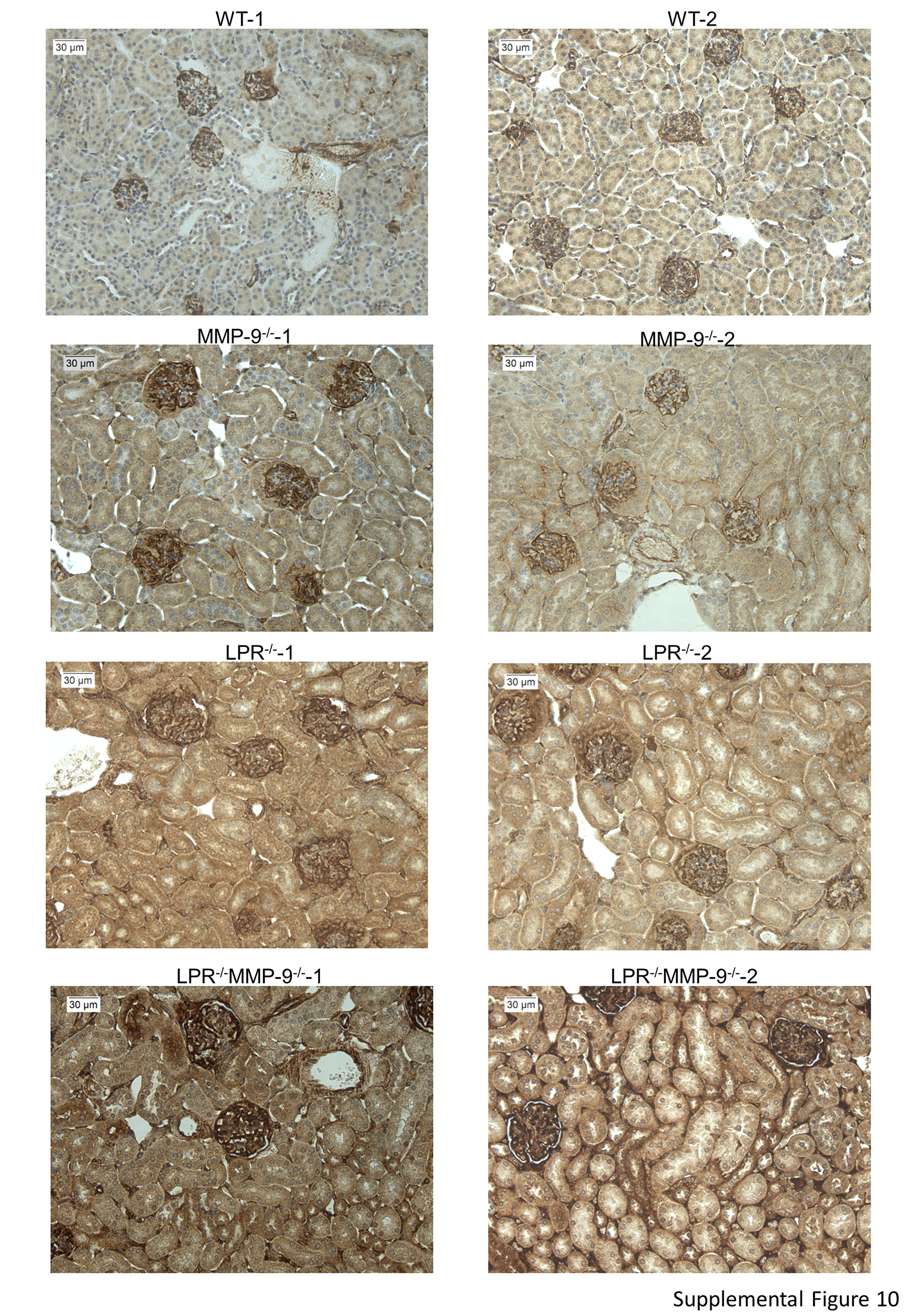

Supplement: Supplemental Figure 10 — Immunohistochemistry analysis (IHC) for C3d of the kidneys of 2 WT mice, 2 MMP-9−/−mice, 2 LPR−/− mice and 2 LPR−/−/MMP-9−/−mice. [file Image_10.TIF]

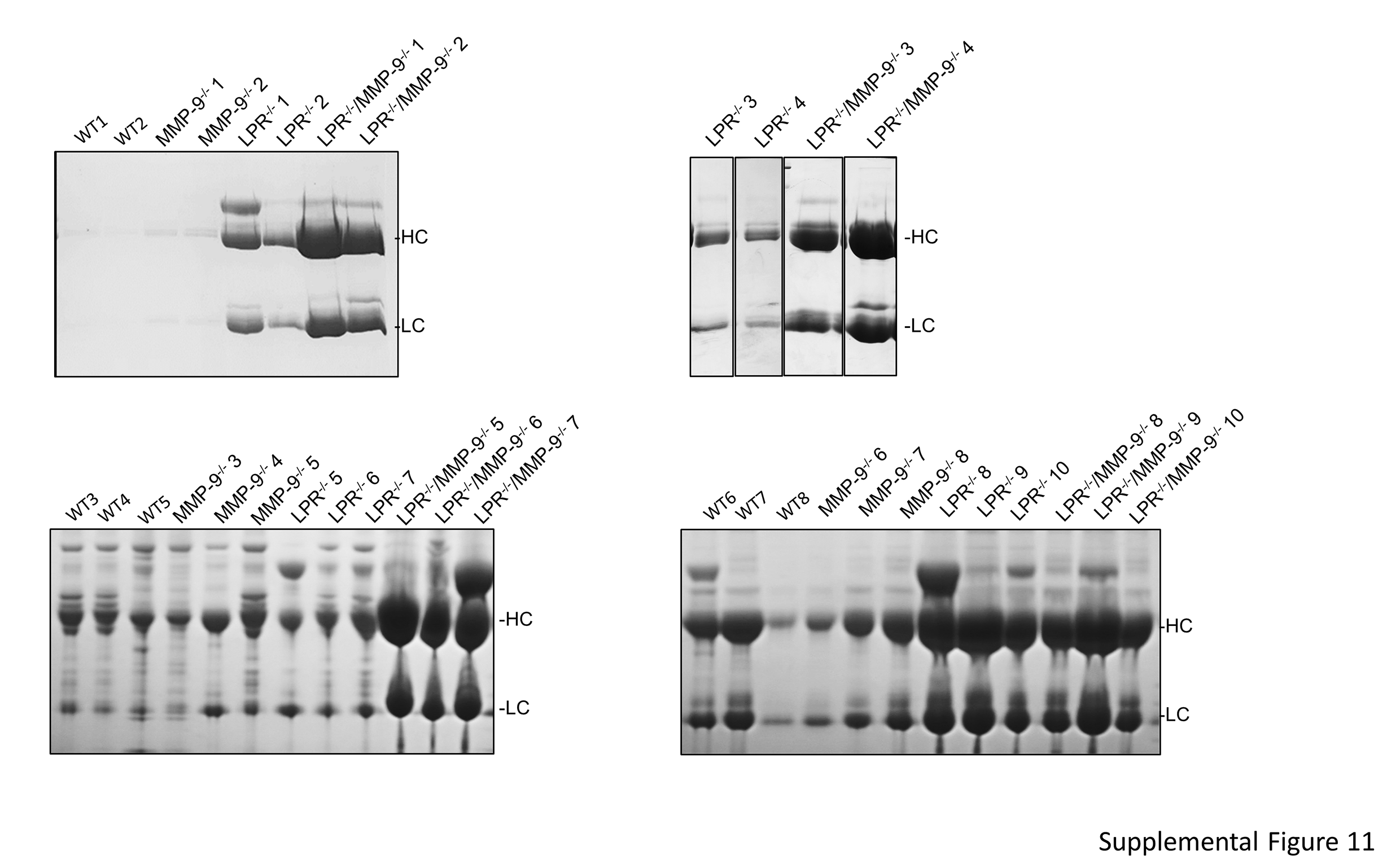

Supplement: Supplemental Figure 11 — Complete set of primary data of purified IC from 8 WT, 8 MMP-9−/−, 10 LPR−/−, and 10 LPR−/−/MMP-9−/− mice, analyzed by SDS-PAGE and Commassie blue staining. The quantification of the heavy chains of these IC has been used to generate the graph shown in Figure 2B. The top left panel is the same as Figure 2A. [file Image_11.TIF]

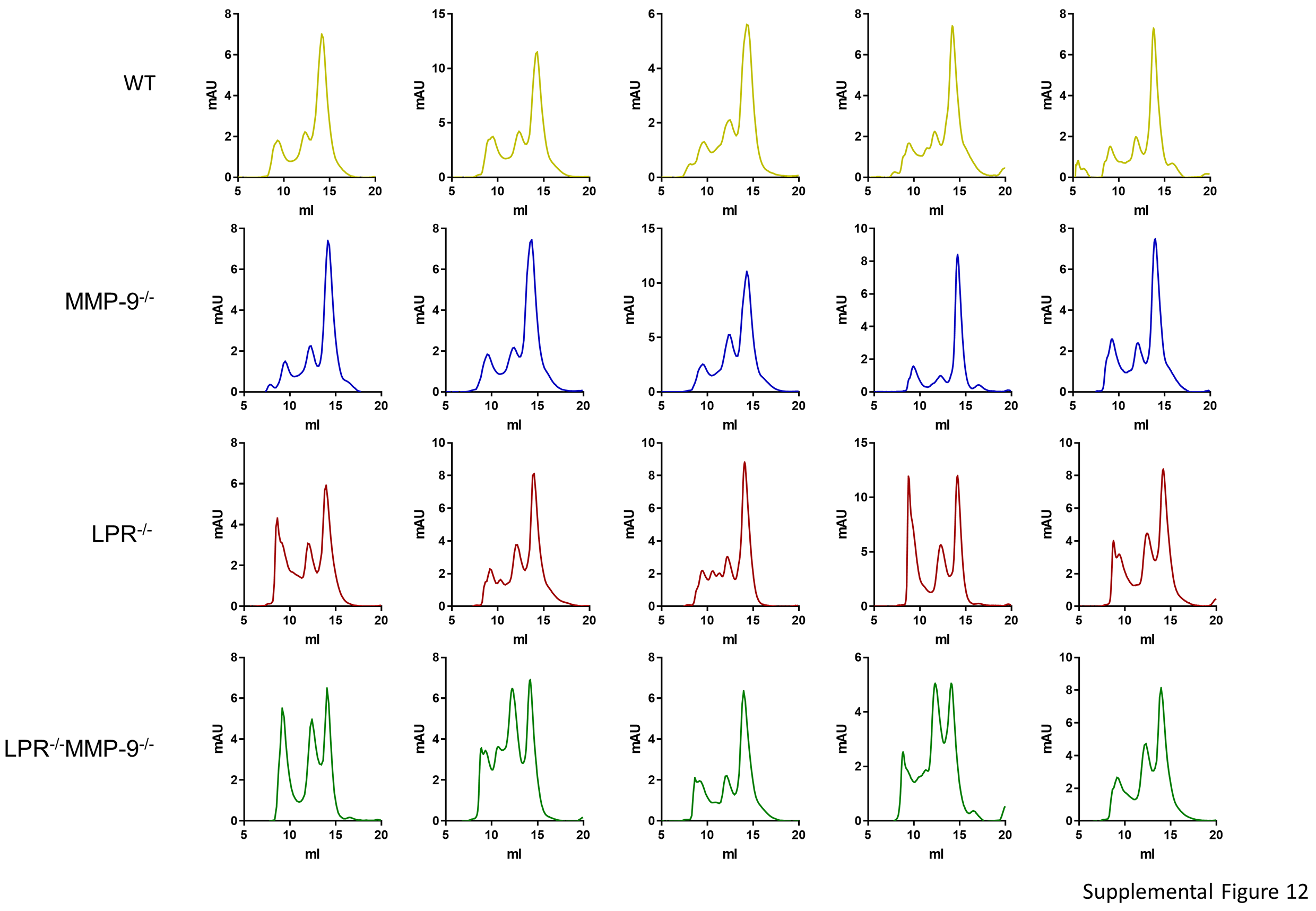

Supplement: Supplemental Figure 12 — Gel filtration analysis profiles of plasma samples from 5 WT (yellow), 5 MMP-9−/− (blue), 5 LPR−/− (red), and 5 LPR−/−/MMP-9−/− (green) mice, used to generate the averaged graph shown in Figure 2D. [file Image_12.TIF]

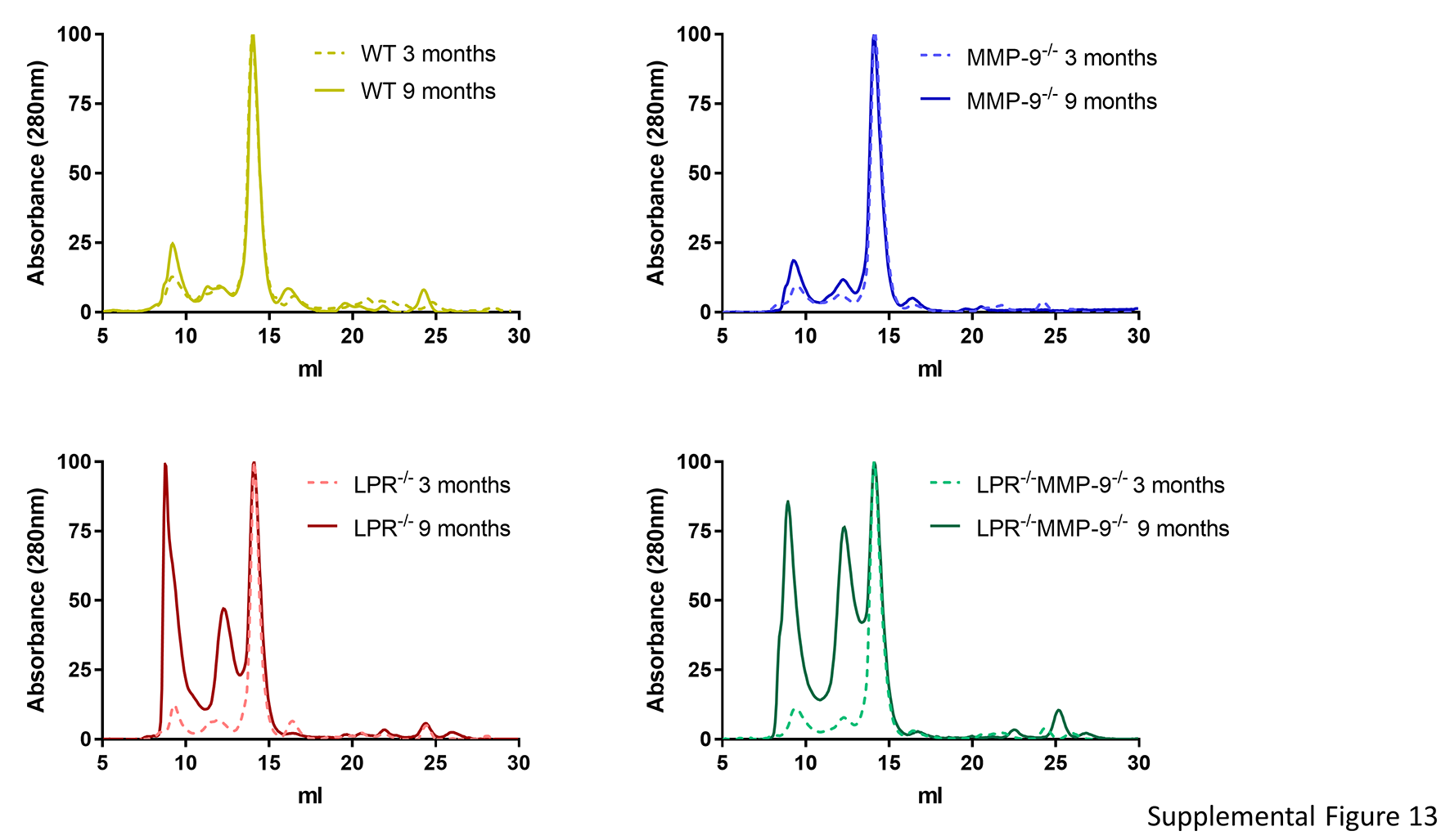

Supplement: Supplemental Figure 13 — Comparisons of gel filtration chromatography profiles from plasma samples at 3 and 9 months for the 4 mouse genotypes. Protein amounts in solutions were determined by absorbance analysis at 280 nm. Representative sequential profiles of one animal for each genotype are provided. [file Image_13.TIF]

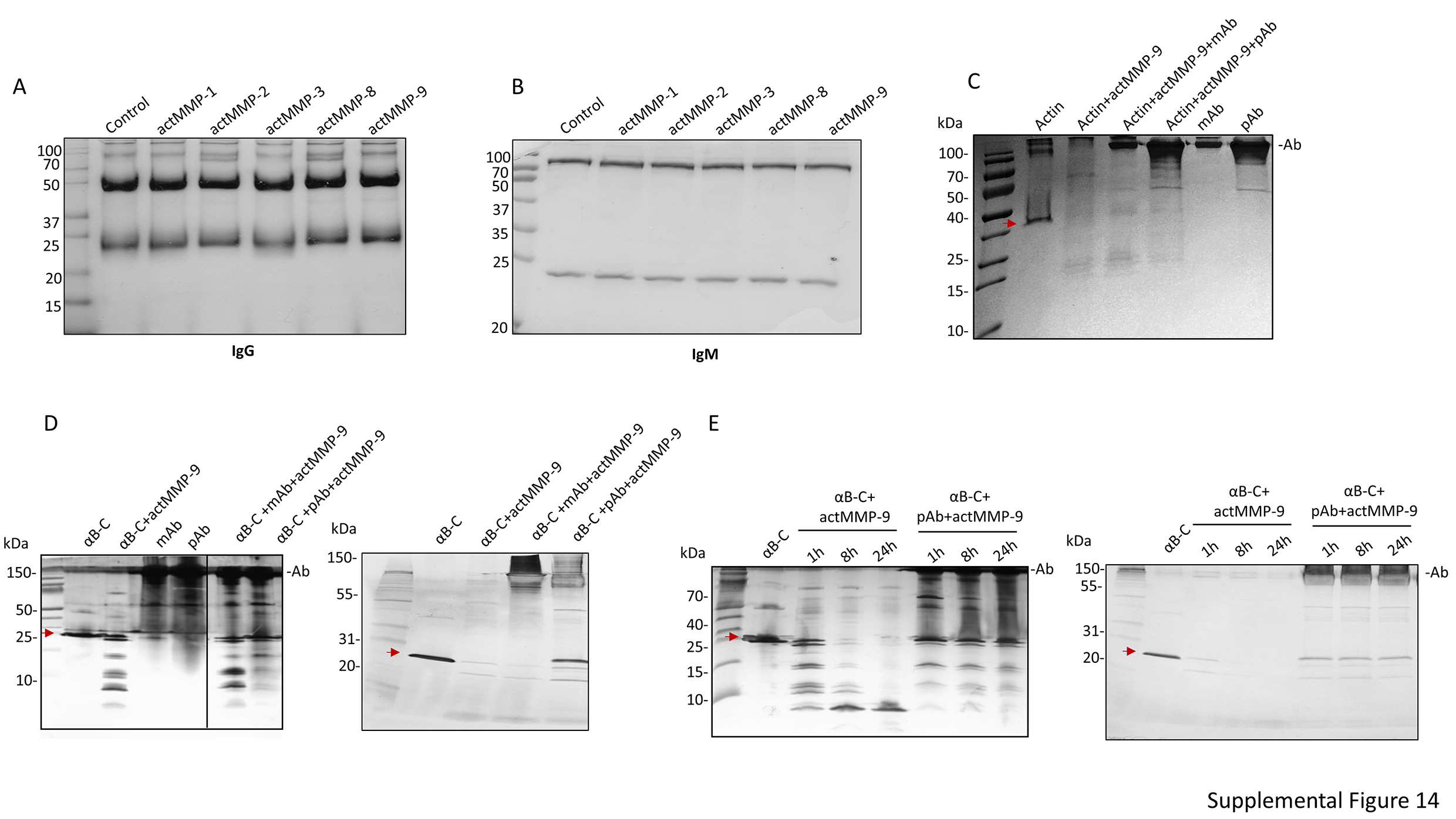

Supplement: Supplemental Figure 14 — Antibodies IgG and IgM and IC of αB-crystallin and actin incubated with actMMP-9. Red arrows indicate the intact autoantigens and substrates of MMP-9. IgG (A) or IgM (B) immunoglobulins were incubated with the indicated active MMPs during 24 h. The proteins in the SDS-PAGE gels were stained with Coomassie Blue. HC, Heavy chain; LC, Light chain. Actin (C) or αB-crystallin (D) in free form or within IC with polyclonal (pAb) or monoclonal antibody (mAb) incubated with actMMP-9 at 37°C. After 24 h the proteins were separated by SDS-PAGE and analyzed by silver staining. (E) Free αB-crystallin (red arrow) or in a pAb-IC incubated with actMMP-9 for 1, 8, or 24 h at 37°C. After incubation, SDS-PAGE separation and silver staining analysis of the proteins were performed. [file Image_14.TIF]

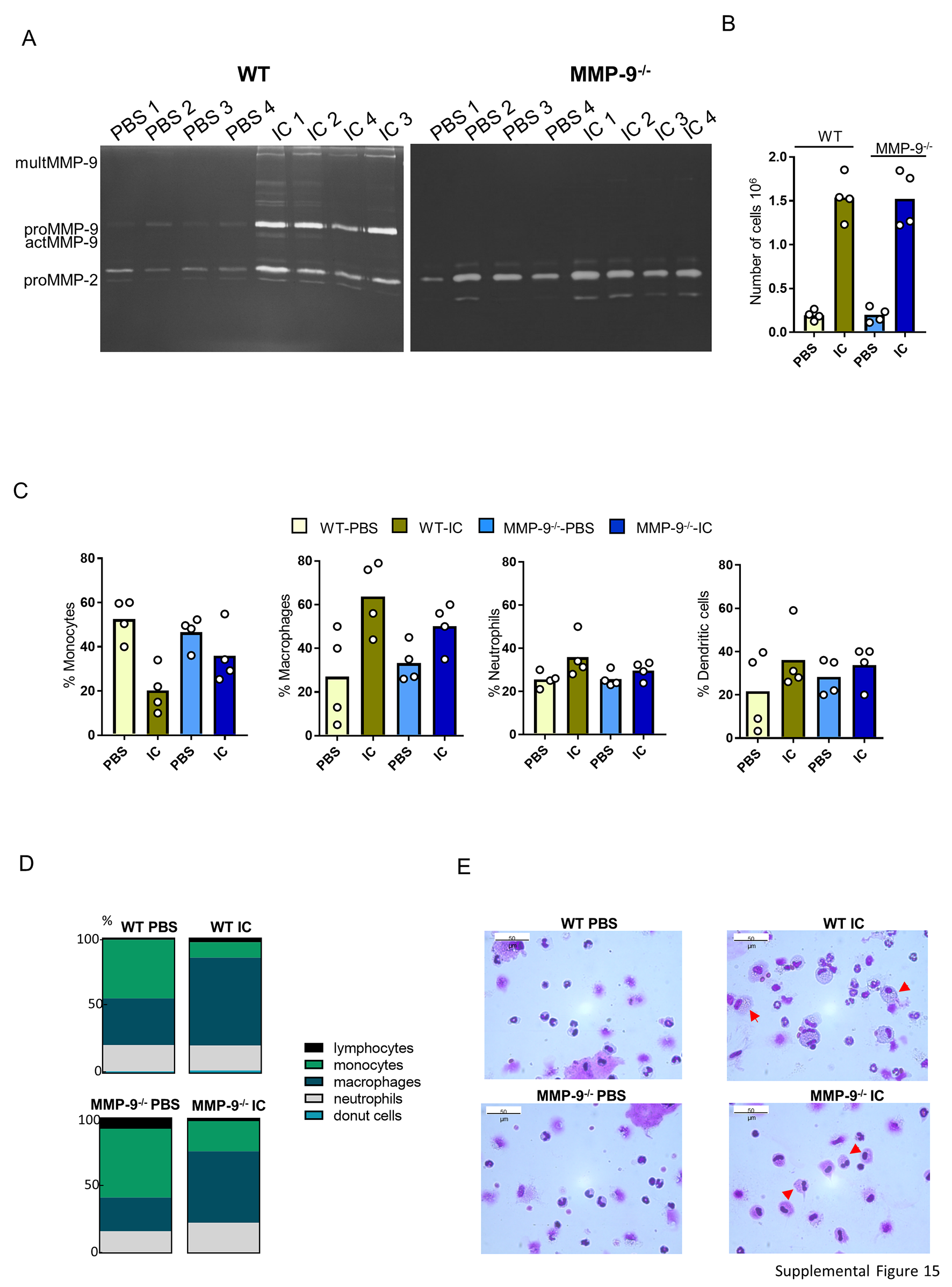

Supplement: Supplemental Figure 15 — Gelatin zymography gel analysis of the exudates obtained after injection of PBS or IC in the air pouch of WT and MMP-9−/− mice and analysis of the cells migrated into the air pouch after PBS or IC injection. (A) Quantification of the bands of proMMP-9, actMMP-9, and MMP-2 was included to generate the graphs shown in Figure 6B. (B) Histograms representing theabsolute numbers of cells collected in the air pouch experiment. (C) Flow cytometry analysis of relative cytospin counts of the lavage exudates from the air pouch after injection of PBS or IC in WT and MMP-9−/− mice. For flow cytometry analysis, monocytes were defined by CD11b and Gr-1, macrophages by CD11b and F4/80, neutrophils by CD11b and Ly6G and dendritic cells by CD11b and CD11c as surface markers. (D) Cytospins were stained with hemacolor, the cells were identified on the basis of their morphology and the relative cell percentages provided as cumulative histograms. Donut cells represent neutrophils with donut-shaped nuclei. The discrimination between monocytes and macrophages was made on the basis of size and presence of vacuoles. (E) Representative images from cytospins of the air pouch lavages after PBS and IC injection in WT and MMP-9−/− mice. [file Image_15.TIF]
