# Supplementary material for: MMP-9/Gelatinase B Degrades Immune Complexes in Systemic Lupus Erythematosus
Source: Front Immunol. 2019 Mar 22;10:538. doi: 10.3389/fimmu.2019.00538 (PMC6440319; doi:10.3389/fimmu.2019.00538)
Supplement: Supplemental Table 1 — Information of the SLE patient cohort of the study. The individual patient numbers (P1-P10) refer to the numbers used throughout the manuscript. [file Table_1.DOCX]

| **Patient** | **Gender** | **Pathology** | **Age**  **(years)** | **Treatment at sampling** | **Clinical disease activity** |
| --- | --- | --- | --- | --- | --- |
| **P1** | Male | SLE, Sjögren | 43 | No documentation | No documentation |
| **P2** | Female | SLE | 46 | Pravasine | non active |
| **P3** | Male | SLE | 25 | Medrol, Plaquenil, Diflucan | non active |
| **P4** | Female | SLE, Sjögren | 51 | Plavix, Emcoretic mitis, Lipitor | non active |
| **P5** | Female | SLE, Sjögren | 38 | Medrol, Plaquenil | non active |
| **P6** | Female | SLE, Sjögren | 50 | Plaquenil, Medrol, Sufrexal, Cacit D3, Pantazol | non active |
| **P7** | Female | SLE | 37 | Plaquenil , Cellcept | active |
| **P8** | Female | SLE | 61 | Prednisone, Plaquenil a, Co-Lisinopril, Asaflow | non active |
| **P9** | Female | SLE | 52 | Medrol, Plaquenil, Ranitidine, Lorazepam, Pulmicort | active |
| **P10** | Female | SLE | 21 | Plaquenil, Triodene | non active |

**Supplemental Table 1:** Information of the SLE patient cohort of the study. The individual patient numbers (P1-P10) refer to the numbers used throughout the manuscript.
