# Supplementary material for: MMP-9/Gelatinase B Degrades Immune Complexes in Systemic Lupus Erythematosus
Source: Front Immunol. 2019 Mar 22;10:538. doi: 10.3389/fimmu.2019.00538 (PMC6440319; doi:10.3389/fimmu.2019.00538)
Supplement: Supplemental Table 2 — Proteins identified by nanoLC-MS/MS peptide sequencing and database search. The numbers (Band 1, 2, 3, and 4) refer to the excised gel slices within the red rectangles in Figure 5C. [file Table_2.DOCX]

| Band 1 | Band 2 | Band 3 | Band 4 |
| --- | --- | --- | --- |
| Apolipoprotein B | Fibronectin | Fibrinogen alpha chain | Fibrinogen alpha chian |
| Lipoprotein A | C1q | Ig heavy mu | MMP-9 |
| C1q | Fibrinogen alpha chain | C4b-binding protein alpha chain |  |
| Ig heavy mu | Complement Factor H | Complement C4 A |  |
|  | C4 B | C1q subcomponent C |  |
|  | Ig heavy mu |  |  |

**Supplemental Table 2:** Proteins identified by nanoLC-MS/MS peptide sequencing and database search. The numbers (Band 1, 2, 3 and 4) refer to the excised gel slices within the red rectangles in Figure 5 panel C.
